# Differential contributions of β-tubulin isotypes to acentrosomal oocyte meiosis in *C. elegans*

**DOI:** 10.1083/jcb.202511199

**Published:** 2026-08-10

**Authors:** Emmanuel T. Nsamba, Anne M. Villeneuve

**Affiliations:** 1 Department of Developmental Biology, Stanford University School of Medicine, Stanford, CA, USA; 2 Department of Genetics, Stanford University School of Medicine, Stanford, CA, USA

## Abstract

Oocyte meiotic spindles must achieve bipolarity and segregate chromosomes in the absence of centrosomes. Here, we use high-resolution immunofluorescence microscopy and live imaging to investigate the differential contributions of β-tubulin isotypes (TBB-1 and TBB-2) to assembly and function of acentrosomal spindles in *Caenorhabditis elegans* oocytes. By combining strains with altered β-tubulin isotype composition with mutations affecting microtubule-crosslinking motor KLP-18 and/or mutations affecting katanin-mediated microtubule severing, we show that TBB-1 and TBB-2 make distinct contributions to promoting spindle bipolarity. Further, by measuring multiple spindle features in wild-type and β-tubulin isotype substitution strains, we reveal contributions of isotype composition to spindle morphology, kinetics of anaphase chromosome separation, and maintenance of spindle structural integrity under stress. Together, our data support a model in which β-tubulin isotype composition helps to maintain a balance between microtubule-crosslinking and severing activities during oocyte meiosis. We further propose that this balance is crucial for establishing spindle bipolarity, maintaining spindle structures, and modulating the dynamics of chromosome separation.

## Teaser

Nsamba and Villeneuve use high-resolution immunofluorescence microscopy and live imaging to investigate *C. elegans* oocyte meiosis, providing evidence for a model in which β-tubulin isotype composition helps to maintain a balance between microtubule-crosslinking and microtubule severing required for normal assembly and function of acentrosomal spindles.

## Introduction

Faithful chromosome segregation during meiosis requires the assembly of bipolar spindles, which physically separate chromosomes through two rounds of divisions to generate haploid gametes. In most metazoan cell types, centriole-containing centrosomes serve as dominant microtubule organizing centers that nucleate and organize spindle microtubules. In contrast, oocytes in most animals, including humans, *Drosophila*, and *Caenorhabditis elegans*, assemble functional spindles through acentrosomal mechanisms—a conserved but mechanistically distinct mode of spindle formation (Mogessie et al., 2018; Mullen et al., 2019). Failure to segregate chromosomes correctly during oocyte meiosis has a profound impact on human health, as it underlies a major fraction of pregnancy loss, infertility, and developmental disorders.

**Figure 1.**
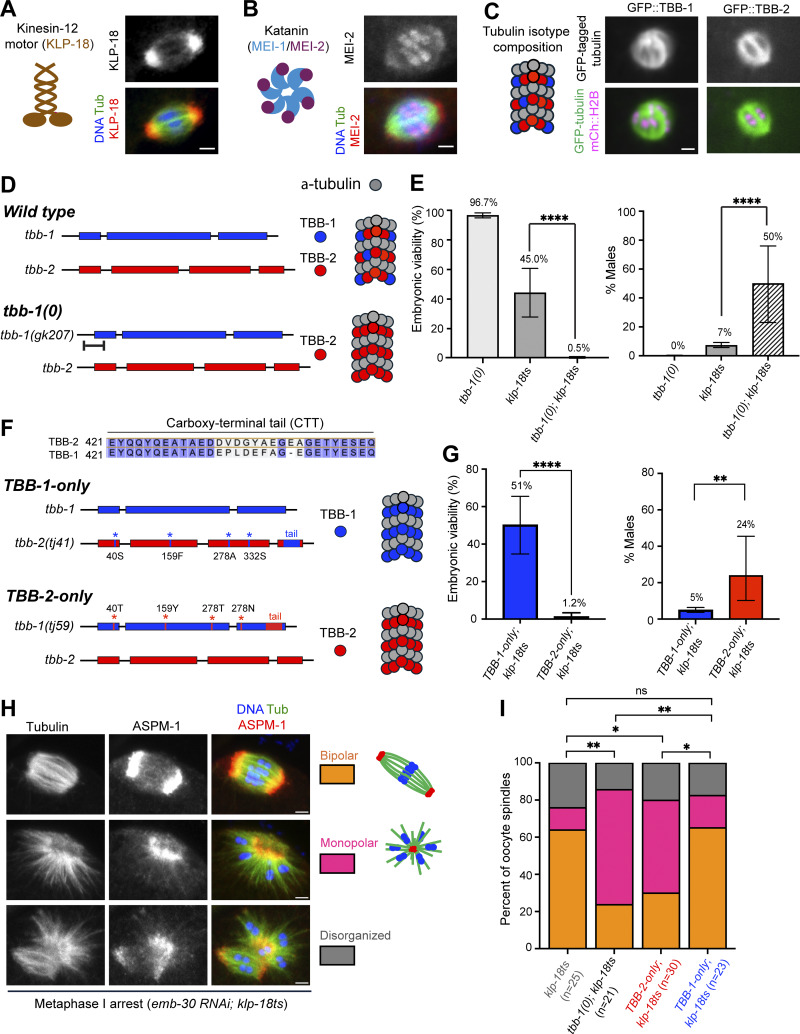
**Differential requirements for β-tubulin isotypes TBB-1 and TBB-2 in promoting bipolarity of oocyte meiotic spindles. (A)** Schematic of plus-end–directed kinesin-12 motor KLP-18 (left) and immunofluorescence image of unarrested wild-type metaphase I oocyte spindle (right), showing KLP-18 localized both at the spindle poles and on microtubules in the midspindle. **(B)** Schematic of microtubule-severing enzyme katanin, comprising six heterodimers of enzymatic subunit MEI-1 and regulatory subunit MEI-2 (left), and immunofluorescence image of unarrested wild-type metaphase I oocyte spindle (right), showing katanin localized both at the spindle poles and near chromosomes at the midspindle. **(C)** Left, depiction of microtubules containing the two β-tubulin isotypes, TBB-1 (blue), and TBB-2 (red). Right, live images of oocyte spindles in worms expressing a GFP-tagged version of the indicated β-tubulin isotype from its endogenous locus, illustrating that both isotypes are incorporated into spindles. **(D)** Diagrams depicting the *tbb-1* and *tbb-*2 gene structures (for the wild-type and *tbb-1* null genotypes), the β-tubulin isotypes produced for each genotype, and the composition of resulting microtubules. **(E)** Graphs reporting frequencies of viable embryos (left) and of male progeny (indicative of X-chromosome mis-segregation, right) produced by hermaphrodites of the indicated genotypes at 20°C (semi-permissive temperature for the *klp-18ts* mutation); these data demonstrate a synthetic genetic interaction between *tbb-1(0)* and *klp-18ts*. For embryo viability graph, error bars indicate SD; for male frequency graph, error bars indicate 95% confidence interval. Data were analyzed using two-tailed Mann–Whitney test and Fisher exact test, respectively; *P < 0.05; **P < 0.01; ****P < 0.0001. **(F)** Top, aligned protein sequences of the carboxy-terminal tails of TBB-1 and TBB-2, where most amino acid differences between the isotypes are located. Bottom, gene structure and microtubule composition diagrams for the β-tubulin isotype substitution strains used in this study, which express the same isotype from both the *tbb-1* and *tbb-2* loci (Honda et al., 2017). To create the *TBB-1-only* strain, Honda et al. used CRISPR engineering to introduce changes into the *tbb-2* locus to code for TBB-1 (*tj41* allele, indicated by blue lines and box); a parallel strategy was used to create the *TBB-2-only* strain, in which the *tbb-1* locus was modified to code for TBB-2. **(G)** Graphs revealing isotype-specific effects on embryo viability and male frequency in the *klp-18ts* mutant background; error bars and P values as in E. **(H)** Immunofluorescence (IF) images of *klp-18ts* spindles arrested at metaphase I (following *emb-30 RNAi*), showing representative examples of the different spindle morphologies observed; scale bars, 2 µm. **(I)** Quantification of spindle morphologies observed in H; *n* = number of spindles. P values were calculated using Fisher exact test with two categories: “bipolar” vs. “monopolar + disorganized.” Significant differences were observed for: *tbb-1(0); klp-18ts* vs *klp-18ts,* P = 0.009; *TBB-2-only; klp-18ts* vs *klp-18ts,* P = 0.015; *TBB-2-only; klp-18ts* vs *TBB-1-only; klp-18ts,* P = 0.014; *tbb-1(0); klp-18ts* vs *TBB-1-only; klp-18ts,* P = 0.008.

*C. elegans* has emerged as a powerful model system for dissecting acentrosomal mechanisms of spindle assembly, as meiotic divisions can be experimentally manipulated and visualized in intact organisms (McNally et al., 2006; Laband et al., 2018; Mullen et al., 2019; Wolff et al., 2022). Pioneering studies established that acentrosomal spindle formation proceeds through a sequence of coordinated steps (Wolff et al., 2016; Chuang et al., 2020) mediated by the dynamic interplay between microtubules and an ensemble of microtubule-interacting factors, including molecular motors and proteins that regulate microtubule dynamics. Central among these factors is the plus-end–directed homodimeric kinesin-12 motor KLP-18, which crosslinks microtubules to establish and maintain spindle bipolarity (Segbert et al., 2003); KLP-18 is localized to spindle poles and the spindle midzone (Fig. 1 A), and its depletion results in monopolar spindles and embryonic lethality (Wignall and Villeneuve, 2009). Also critical is the microtubule-severing enzyme katanin, comprising the MEI-1 catalytic subunit and MEI-2 regulatory subunit in *C. elegans* (Mains et al., 1990; Clark-Maguire and Mains, 1994), which is localized to spindle poles and around chromosomes in the spindle midzone (Fig. 1 B; and McNally et al., 2006; Srayko et al., 2000). Katanin functions as a central orchestrator of multiple aspects of acentrosomal spindle assembly in *C. elegans*, contributing to spindle pole formation and organization, chromosome alignment, and regulation of microtubule length and dynamics (Srayko et al., 2000; Srayko et al., 2006; Connolly et al., 2014; McNally et al., 2014; Lantzsch et al., 2021).

The coordinated activities of KLP-18, katanin, and other microtubule-interacting proteins during acentrosomal spindle assembly necessitate their productive engagement with microtubules that are compositionally heterogeneous, containing multiple variants, or isotypes, of α- and β-tubulin subunits (Fig. 1 C). Accumulating evidence across diverse systems indicates that tubulin isotypes confer distinct functional properties to microtubule-dependent processes (Nsamba and Gupta, 2022). During mitosis in budding yeast, for example, α-tubulin isotypes exhibit specialization for distinct spindle positioning mechanisms (Nsamba et al., 2021) and contribute divergent properties that balance anaphase spindle dynamics (Nsamba et al., 2025). Similarly, studies in *C. elegans*, *Drosophila*, and mice have revealed isotype-specific contributions to thermal tolerance (Pallotto et al., 2022; Myachina et al., 2017), neurite growth (Lockhead et al., 2016; Zheng et al., 2017; Baran et al., 2010), cilia function (Silva et al., 2017; Hurd et al., 2010), spermatogenesis (Hutchens et al., 1997; Hoyle and Raff, 1990), and neuronal development (Bittermann et al., 2019; Buscaglia et al., 2020) and axonal regeneration (Latremoliere et al., 2018).

**Figure S1.**
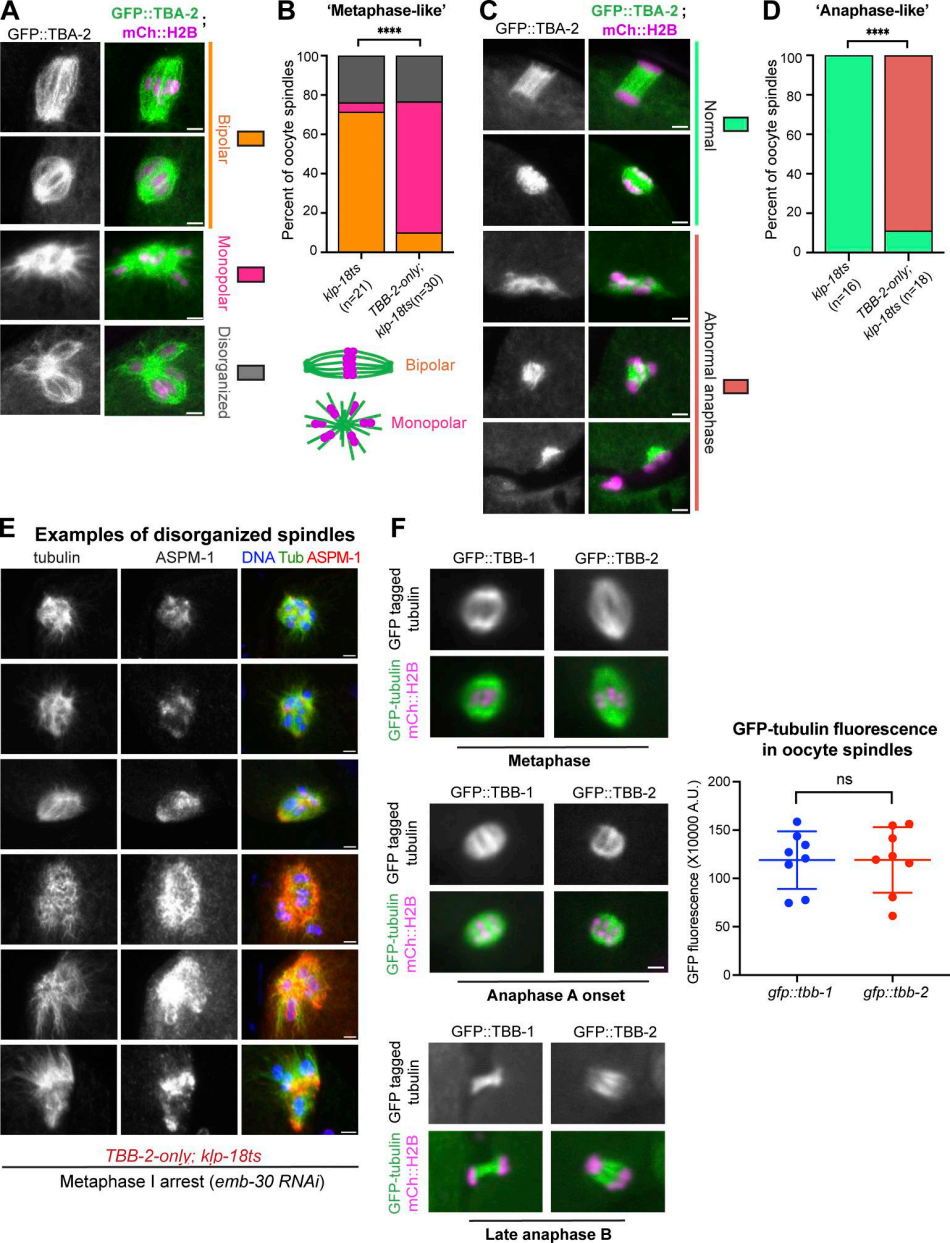
**Loss of TBB-1 exacerbates meiotic spindle assembly defects caused by *klp-18ts*. (A–D)** Analysis of spindle morphologies in unarrested meiotic embryos harboring the *klp-18ts* mutation. **(A)** Representative images of GFP and mCherry fluorescence in unarrested metaphase/metaphase-like spindles in fixed *klp-8ts* single mutant and *TBB-2-only; klp-8ts* double mutant embryos expressing GFP::TBA-2 and mCherry::H2B. Monopolar or disorganized spindles were classified as “metaphase-like” if bivalents were unseparated and were flanked by discernable microtubule bundles. **(B)** Quantification of spindle morphologies observed in A. Similar to data from immunofluorescence images of metaphase I–arrested spindles (Fig. 1, H and I), loss of TBB-1 exacerbates spindle bipolarity defects in the *klp-18ts* mutant background. Analysis of spindle bipolarity as in Fig. 1 I, P = 0.0001. **(C)** Representative GFP::TBA-2 and mCherry::H2B fluorescence images showing anaphase-like spindle morphologies in fixed *klp-8ts* single mutant and *TBB-2-only; klp-8ts* double mutant embryos. Anaphases were scored as normal based on the presence of two separated chromosome masses of comparable size flanking a dense microtubule array. Spindles were classified as “abnormal anaphase” if they contained >2 chromosome masses of distinct sizes at the periphery of a dense microtubule array, a single chromosome mass adjacent to a compact dense microtubule array, or a disorganized set of chromosome masses within a condensed spindle lacking a central dense microtubule array. **(D)** Quantification of anaphase spindle morphologies in C. *TBB-2-only; klp-8ts* embryos exhibit defects in anaphase spindle assembly and aberrant chromosome segregation likely arising from earlier defects in spindle bipolarity. In Fig. S1, B and D, both meiosis I and meiosis II spindles were grouped together. Scale bars in all images = 2.0 μm. **(E)** Representative IF images of disorganized metaphase I–arrested spindles observed in the *TBB-2-only; klp-18ts* mutant background. In contrast to monopolar or bipolar spindles, which have one or two poles marked by ASPM-1 foci, respectively, spindles classified as disorganized may exhibit no ASPM-1 foci (apolar), more than two ASPM-1 foci (multipolar), or have ASPM-1 distributed broadly within the spindle. Scale bars, 2.0 µm. **(F)** Left, representative live images of oocyte spindles expressing endogenously tagged β-tubulins (either GFP::TBB-1 or GFP::TBB-2), showing that TBB-1 and TBB-2 are both readily incorporated into oocyte meiotic spindles. Right, accompanying quantification of live GFP fluorescence in prometaphase/metaphase spindles. ns, not significant; ****P < 0.0001; IF, immunofluorescence.

The *C. elegans* system, which encodes two germline-expressed β-tubulin isotypes, TBB-1 and TBB-2 (Nishida et al., 2021) provides an outstanding opportunity to investigate the functional significance of tubulin isotype diversity to the formation and function of acentrosomal meiotic spindles. β-tubulin isotype substitution strains have been created that express wild-type levels of tubulin but produce only one of the two isotypes, permitting direct investigation of potential functional differences (Honda et al., 2017). At a superficial level, these two β-tubulin isotypes appear at least partially functionally redundant, as embryos from the tubulin isotype substitution strains are fully viable. Moreover, TBB-1 and TBB-2 are both readily incorporated into wild-type oocyte spindles throughout the meiotic cell cycle (Fig. 1 C and Fig. S1). Despite this apparent redundancy, genetic analyses involving mutations that either partially reduce or cause ectopic persistence of katanin activity have provided evidence suggesting that the microtubules containing the TBB-2 β-tubulin isotype are preferred substrates for katanin-mediated severing *in vivo* (Lu et al., 2004). However, the extent of functional specialization of the TBB-1 and TBB-2 isotypes and their specific contributions to meiotic spindle assembly and function have remained largely unexplored.

Here, we employ high-resolution immunofluorescence microscopy and live imaging in *C. elegans* strains with altered β-tubulin isotype composition to investigate how TBB-1 and TBB-2 differentially contribute to acentrosomal spindle assembly and chromosome segregation dynamics. Through a sensitized genetic approach, we provide evidence that TBB-1 and TBB-2 make distinct contributions to mechanisms that enable stable formation of bipolar meiotic spindles. Further, by comparing multiple features of meiotic spindles from wild-type and β-tubulin isotype substitution strains, we reveal hidden contributions of isotype composition to spindle length, katanin localization, and kinetics of anaphase chromosome separation. We interpret our data in the context of a model in which β-tubulin isotype helps to counterbalance the effects of microtubule-crosslinking and microtubule severing activities, thereby promoting normal assembly and function of acentrosomal meiotic spindles.

## Results

### Absence of the TBB-1 isotype exacerbates oocyte meiotic defects caused by *klp-18ts*

To test whether a sensitized genetic background might be useful for revealing potential differential contributions of β-tubulin isotypes to assembly and/or function of meiotic spindles, we employed *klp-18(or447ts)*, a temperature-sensitive missense mutation causing partial loss-of-function of KLP-18 (Connolly et al., 2014), a plus-end–directed kinesin-12 microtubule-crosslinking motor required for bipolarity of oocyte meiotic spindles (Segbert et al., 2003; Wignall and Villeneuve, 2009). The *klp-18ts* mutant provides a valuable dynamic range for this approach, exhibiting near-normal embryonic viability (reflecting normal meiosis) at 15°C and nearly complete embryonic lethality and abnormal meiosis at 25°C, with an intermediate phenotype at the semi-permissive temperature of 20°C. Through construction of double mutants, we discovered a synthetic genetic interaction between a *tbb-1(0)* null mutation and *klp-18(or447ts)* at 20°C (Table S1; and Fig. 1, D and E). Whereas *tbb-1(0)* and *klp-18ts* single mutants exhibited 97% and 45% embryonic viability, respectively, nearly all embryos produced by *tbb-1(0); klp-18ts* double mutant animals were inviable (0.5% viability; P < 0.0001 compared with either single mutant). Further, 50% of the rare surviving progeny were XO males (compared with 0.1% and 7% for the respective single mutants; P < 0.0001), a phenotype reflecting missegregation of sex chromosomes during meiosis in XX hermaphrodites.

To further investigate this synthetic interaction, we introduced the *klp-18ts* mutation into β-tubulin substitution strains generated by CRISPR editing to enable expression of single isotypes at normal protein levels (Honda et al., 2017). “*TBB-1-only*” strains express TBB-1 from both the *tbb-1* and *tbb-2* loci, while “*TBB-2-only*” strains express TBB-2 from both loci, thus maintaining normal total β-tubulin levels and supporting normal embryonic viability while altering isotype composition of the microtubules (Honda et al., 2017; Fig. 1 F; and Table S1). We found that *TBB-1-only; klp-18ts* double mutant worms exhibited levels of embryonic viability and incidence of male progeny similar to those observed for the *klp-18ts* single mutant (Table S1 and Fig. 1 G). In contrast, *TBB-2-only; klp-18ts* double mutant worms exhibited severe synthetic defects comparable with the original *tbb-1; klp-18ts* synthetic interaction: 1.2% embryo viability and 24% male progeny (Table S1 and Fig. 1 G). Together, these data suggest that the presence of TBB-1 in microtubules is required for oocyte meiotic spindle function when KLP-18 activity is partially impaired.

To understand the cellular basis for these genetic interactions, we used immunofluorescence microscopy to directly assess the morphology of metaphase I–arrested oocyte meiotic spindles (Furuta et al., 2000), using antibodies against ASPM-1 as a spindle pole marker (Wignall and Villeneuve, 2009) and anti–α-tubulin antibodies to visualize microtubules (Fig. 1, H and I; and Fig. S1 E). Whereas the majority of spindles in the *klp-18ts* single mutant and the *TBB-1-only; klp-18ts* double mutant exhibited normal bipolar organization (similar to Fig. 1 A), double mutants lacking the TBB-1 isotype (both *tbb-1(0); klp-18ts* and *TBB-2 only; klp-18ts*) exhibited elevated frequencies of monopolar spindles. Analysis of meiotic spindles in unarrested embryos similarly revealed an elevated incidence of monopolar spindles in the *TBB-2-only; klp-18ts* double mutant relative to the *klp-18ts* single mutant (Fig. S1, A–D). We conclude that microtubules containing only the TBB-2 β-tubulin isoform are deficient in promoting bipolarity of acentrosomal oocyte meiotic spindles under conditions where KLP-18–dependent microtubule-crosslinking activity is compromised.

**Figure S2.**
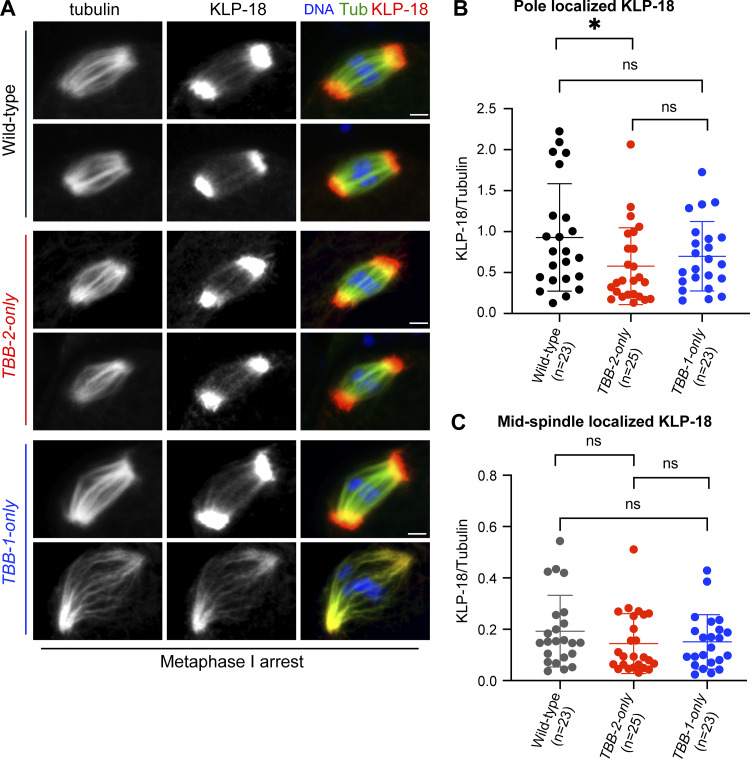
**Lack of evidence for a difference in KLP-18 localization between TBB-1-only and TBB-2-only oocyte meiotic spindles. (A)** Representative IF images of metaphase I–arrested oocyte spindles showing KLP-18 pole and midzone localization in the indicated genotypes. Scale bar = 2.0 μm. **(B and C)** Quantification of KLP-18 IF signals normalized to tubulin IF signals at the spindle poles (B) and in the spindle midzone (C). Error bars indicate SD. *n* = total number of embryos analyzed per genotype. Data were analyzed using two-tailed Mann–Whitney test; ns, not significant; *P < 0.05. IF, immunofluorescence.

In principle, worsening of the *klp-18* mutant phenotype could potentially be caused by reduced recruitment of the mutant KLP-18 protein. However, immunostaining experiments did not detect differences in either midzone or pole-associated KLP-18 signals between *TBB-1-only* and *TBB-2-only* spindles (Fig. S2, A–C), suggesting that impaired KLP-18 recruitment is unlikely to be responsible for the observed differences in genetic sensitivity to the *klp-18ts* mutation.

### Mutations causing either reduced sensitivity to or reduced activity of katanin suppress the synthetic meiotic defects of *TBB-2-only; klp-18ts*

Based on the prior work of Lu et al. (2004), we hypothesized that the synthetic meiotic defects observed in the *TBB-2-only; klp-18ts* double mutant might result from elevated sensitivity of TBB-2-only microtubules to the microtubule severing enzyme katanin. We tested this hypothesis in two ways.

**Figure 2.**
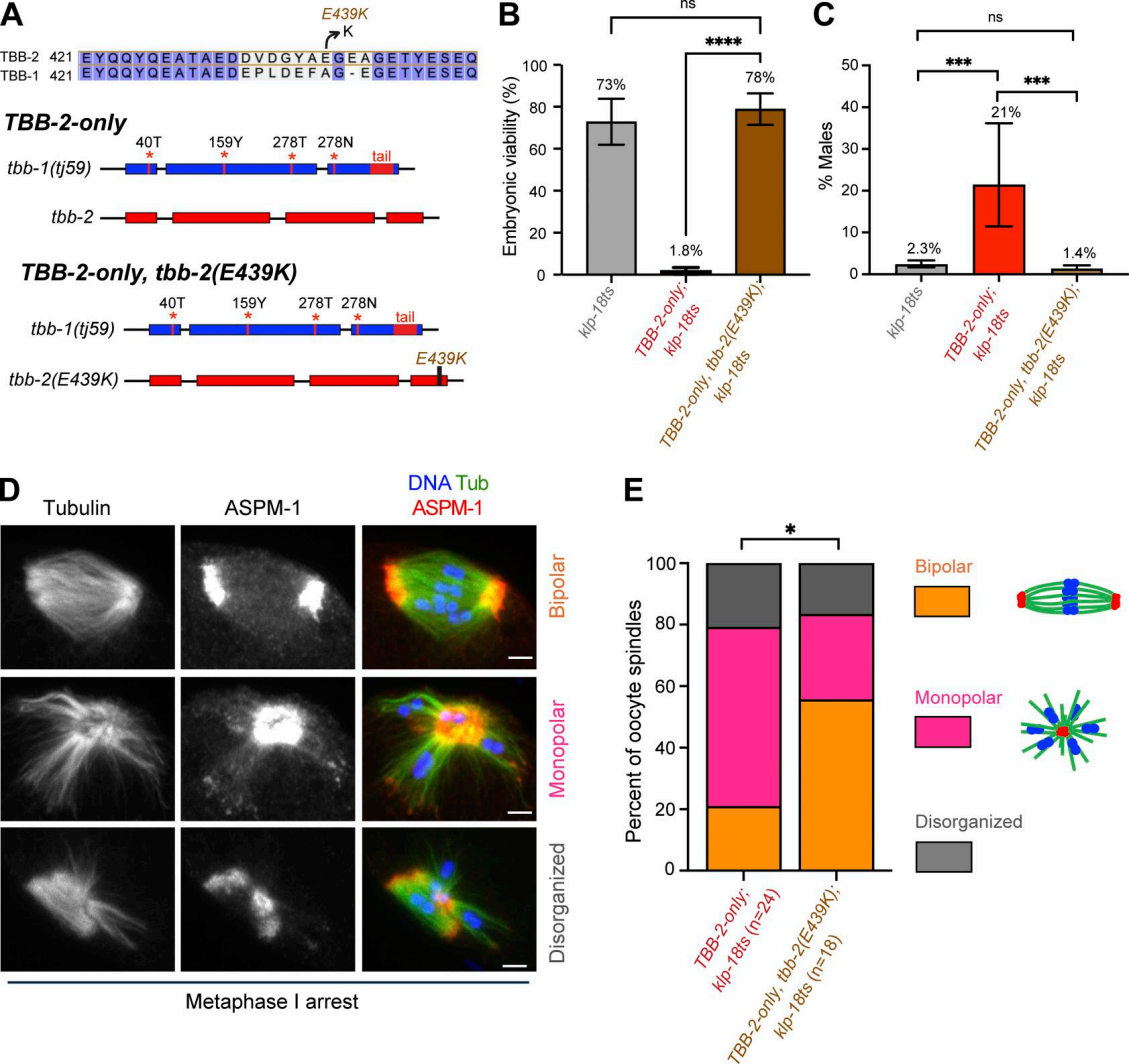
**The *tbb-2(E439K)* mutation, inferred to confer partial resistance to katanin-mediated severing, suppresses synthetic meiotic defects of *TBB-2-only; klp-18ts*. (A)** Top, protein sequence alignment showing E439K position in TBB-2 carboxy-terminal tail. Bottom, gene structure diagrams showing the location of the *tbb-2(E439K)* missense mutation in the *tbb-2* locus. The *TBB-2-only, tbb-2(E439K)* strain expresses wild-type TBB-2 from the *tbb-1* locus and mutant TBB-2(E439K) from the *tbb-2* locus. **(B and C)** Quantification of embryonic viability and male frequency at 20°C, as in Fig. 1 E. ns, not significant; *P < 0.05; ***P < 0.001; ****P < 0.0001. **(D)** Representative IF images of metaphase I arrested oocyte spindles in the *tbb-2(E439K)* mutant background; scale bars, 2 µm. **(E)** Quantification of spindle phenotypes observed in D; *n* = number of spindles. Analysis of spindle bipolarity was performed as in Fig. 1 I, *P = 0.048. Since between-experiment variability is expected for experiments involving temperature-sensitive mutations at semi-permissive conditions, all graphs present data for a set of genotypes that were analyzed in parallel. We note that 75% bipolar spindles were observed for *TBB-2-only, tbb-2(E439K)* in Fig. 3 D. IF, immunofluorescence.

First, we used a missense mutation, *tbb-2(E439K)*, which was initially isolated based on suppression of lethality caused by ectopic persistence of katanin activity in cleavage-stage embryos and is inferred to confer partial resistance of microtubules to katanin-mediated severing (Lu et al., 2004). We recreated the *tbb-2(E439K)* mutation in a wild-type background and confirmed that it does not impair embryonic viability (Table S2). Moreover, we found that introduction of *tbb-2(E439K)* into the *TBB-2-only; klp-18ts* background (so that the strain expresses wild-type TBB-2 from the *tbb-1* locus and mutant TBB-2(E439K) from the *tbb-2* locus; Fig. 2 A) was indeed sufficient to suppress the embryonic lethality, sex chromosome missegregation, and meiotic spindle defects observed in the *TBB-2-only; klp-18ts* double mutant (Table S2; and Fig. 2, B–E).

**Figure 3.**
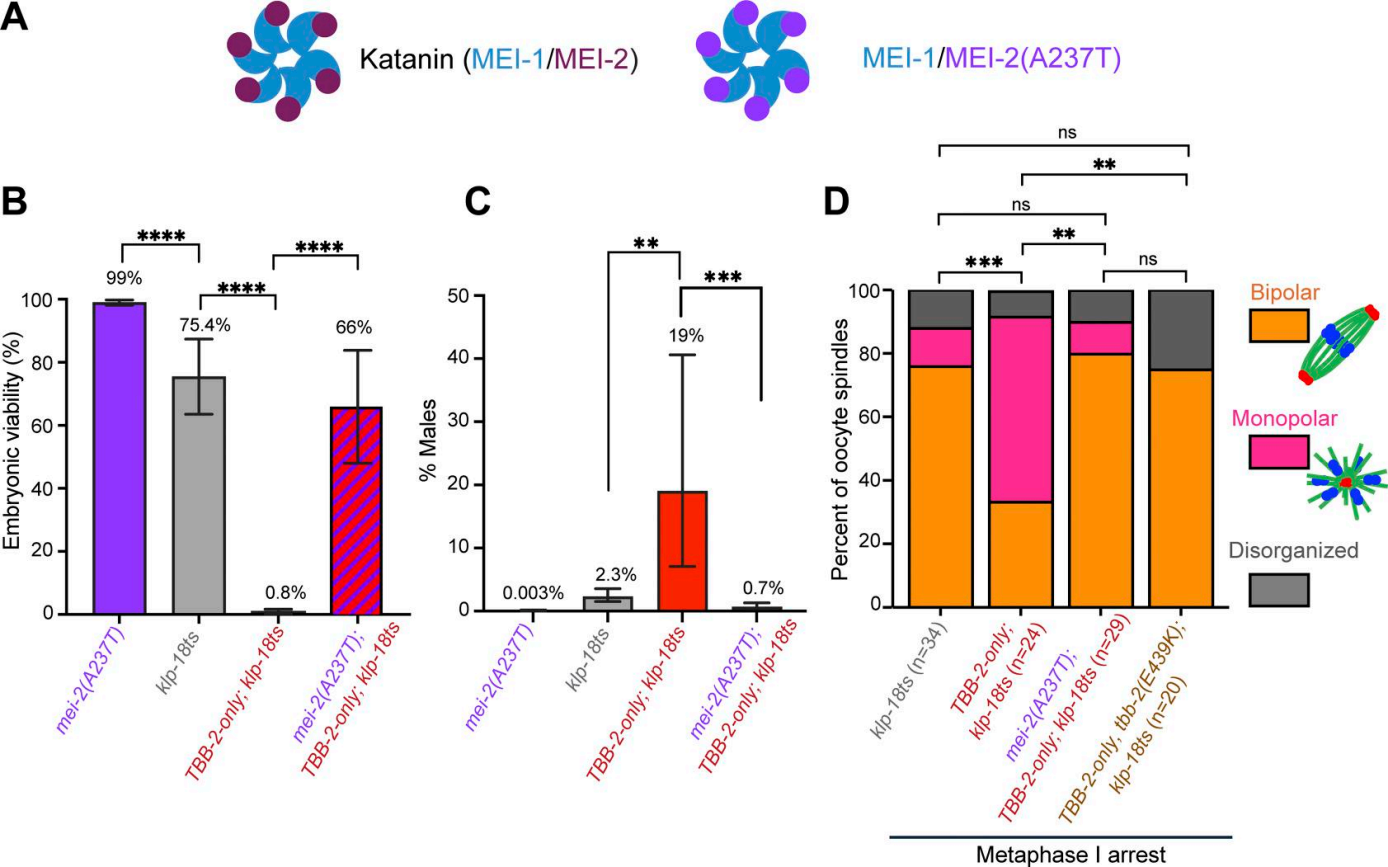
**Reduction of katanin activity suppresses synthetic meiotic defects of *TBB-2-only; klp-18ts.* (A)** Schematic of katanin in wild type and in the *mei-2(A237T)* partial loss-of-function mutant (Srayko et al., 2000). **(B and C)** Quantification of embryonic viability and male frequency at 20°C, as in Fig. 1 E. ns, not significant; **P < 0.01; ***P < 0.001; ****P < 0.0001. *mei-2(A237T)* restores embryonic survivability and suppresses chromosome missegregation of *TBB-2 only; klp-18ts*. **(D)** Quantifications of morphologies observed in IF images of metaphase I arrested oocyte spindles; *n* = number of spindles. Analysis of spindle bipolarity as in Fig. 1 I. Significant differences were observed for: *TBB-2-only; klp-18ts* vs *klp-18ts*, P = 0.001; *TBB-2-only; klp-18ts* vs *tbb-2(E439K),TBB-2-only; klp-18ts,* P = 0.003; *TBB-2-only;klp-18ts* vs *mei-2(A437T); TBB-2-only; klp-18ts*, P = 0.002. IF, immunofluorescence.

Second, we sought to test whether reducing katanin activity itself might similarly suppress the *TBB-2-only; klp-18ts* meiotic defects. For this purpose, we used *mei-2(A237T),* a partial loss-of-function allele affecting katanin accessory subunit MEI-2 (Srayko et al., 2000) that was previously shown to reduce katanin severing activity *in vitro* without affecting embryonic viability (McNally et al., 2014) (Table S3 and Fig. 3 A). Introduction of the *mei-2(A237T)* missense mutation into the *TBB-2-only; klp-18ts* mutant background provided robust suppression comparable with that observed with *tbb-2(E439K)* (Table S3; and Fig. 3, B–D). Critically, *mei-2(A237T)* substantially restored bipolar spindle formation to levels similar to those observed for the *klp-18ts* single mutant.

Together, our data showing suppression both by reducing sensitivity of microtubules to katanin and by reducing katanin activity support our hypothesis that the enhanced meiotic defects observed in *TBB-2-only; klp-18ts* oocytes reflect hypersensitivity of TBB-2-only microtubules to katanin-mediated severing. Our data are consistent with the interpretation that β-tubulin isotype composition promotes bipolarity of *C. elegans* oocyte meiotic spindles in part by modulating susceptibility of spindle microtubules to katanin-mediated severing.

### β-tubulin isotype composition differentially affects association of katanin with meiotic spindles

**Figure 4.**
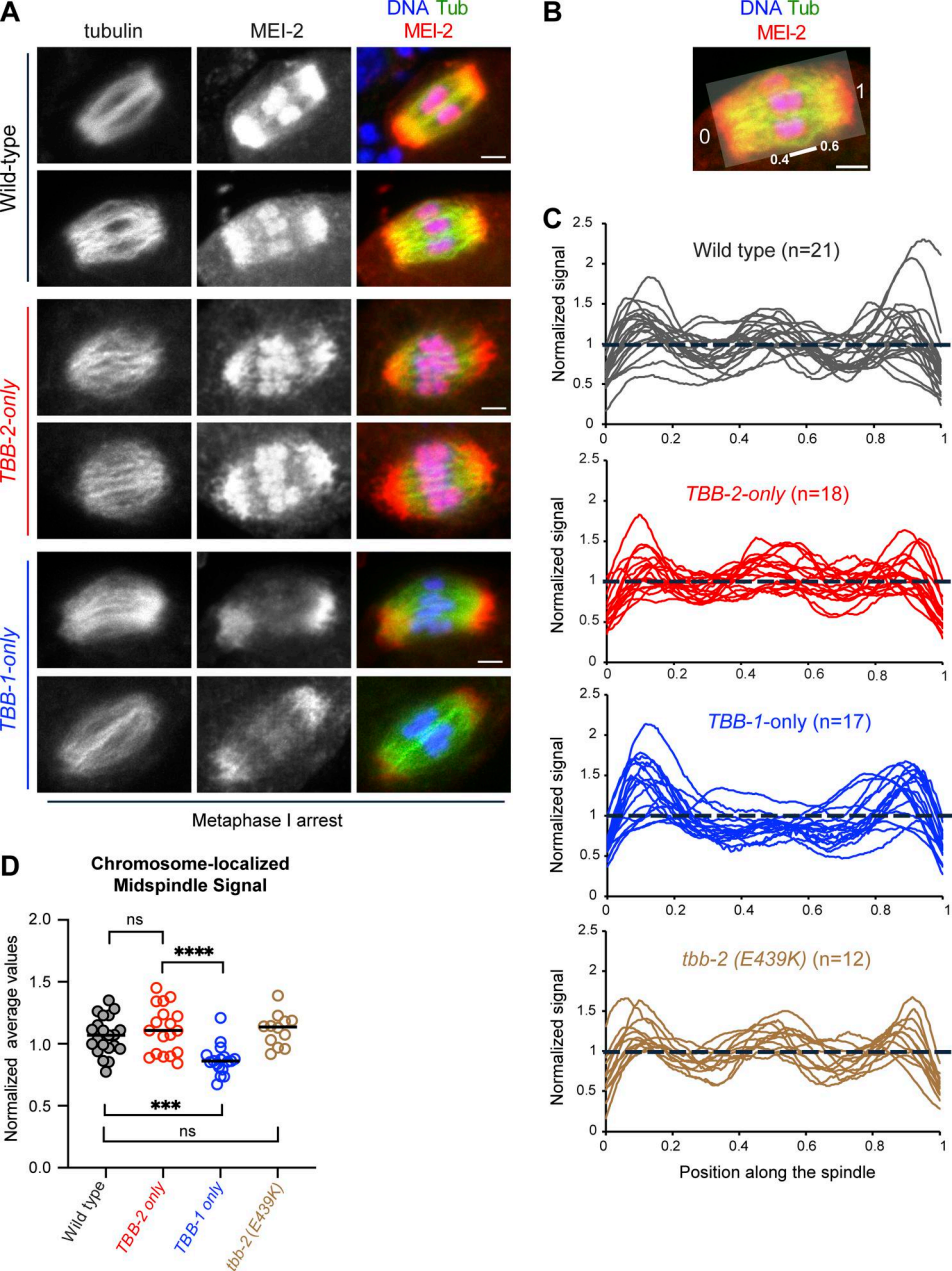
**β-tubulin isotype composition differentially affects association of katanin with meiotic spindles. (A)** Immunolocalization of MEI-2 at the poles and around the chromosomes in metaphase I–arrested oocyte spindles of the indicated genotypes. Images illustrate the range of patterns observed; scale bars = 2.0 μm. **(B)** Depiction of scheme for quantifying the relative MEI-2 distribution along the spindle axis (from 0 to 1), using a line-tracing method wherein the width of the line corresponds to the full width of the spindle (see Materials and methods). The region from 0.4 to 0.6 represents the central portion of the spindle in the vicinity of chromosomes. **(C)** Pileups of individual line traces of normalized MEI-2 signal intensity from measurements conducted as in B (and Materials and methods). (The *tbb-2(E439K)* strain is wild-type at the *tbb-1* locus). **(D)** Quantification of the normalized average MEI-2 signal in the chromosome-occupied central spindle (positions 0.4–0.6); each data point represents an individual spindle. Error bars indicate SD. MEI-2 signal is significantly depleted in central spindles (relative to the poles) in *TBB-1-only.* Data were analyzed using two-tailed Mann–Whitney test; ns, not significant; ***P < 0.001; ****P < 0.0001. Similar patterns were observed for MEI-1 (Fig. S3).

**Figure S3.**
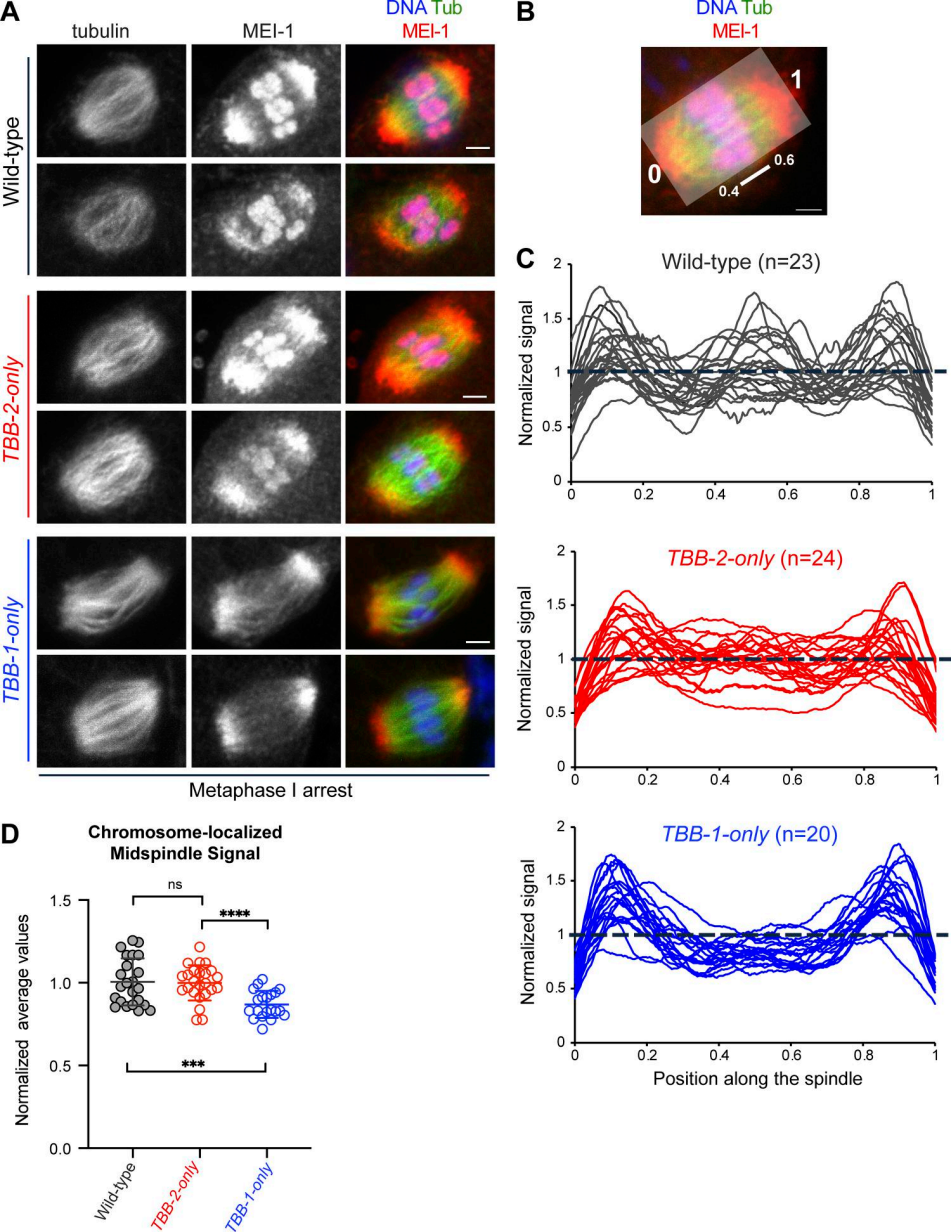
**TBB-1-only spindles exhibit reduced localization of katanin around chromosomes relative to the poles. (A)** MEI-1 immunofluorescence in metaphase I–arrested oocytes of the indicated genotypes. Scale bar = 2.0 μm. **(B)** Analysis schematic for measuring MEI-1 distribution across spindle axis. Positions 0.4–0.6 represents central region in the vicinity of chromosomes. **(C)** Pileups of individual line traces of normalized MEI-1 signal intensity from measurements conducted as in B from spindles of the indicated genotypes. **(D)** Quantification of the normalized average MEI-1 signal in the chromosome-occupied central spindle (positions 0.4–0.6); each data point represents an individual spindle. Error bars indicate SD. Data were analyzed using two-tailed Mann–Whitney test; ns, not significant; ***P < 0.001; ****P < 0.0001.

To further investigate the relationship between β-tubulin isotype composition and katanin, we examined the localization of MEI-2 (katanin regulatory subunit) and MEI-1 (katanin enzymatic subunit) in metaphase I–arrested oocyte spindles from wild-type, *TBB-1-only*, and *TBB-2-only* strains (in a wild-type *klp-18(+)* background [Fig. 4 and Fig. S3]). Previous studies showed that katanin localizes to *C. elegans* oocyte spindle poles, where it promotes pole organization, and also localizes with chromosomes in the middle of the spindle, although the functional significance of chromosome-localized katanin is unclear (Srayko et al., 2000; McNally et al., 2006). We similarly observed spindle pole localization of MEI-2 and MEI-1 in all genotypes, but we also noted that *TBB-1-only* oocyte spindles appeared to have reduced chromosome-localized MEI-2 and MEI-1 in their central spindles relative to their poles.

We conducted line profile analyses to quantitatively evaluate the spatial distribution of MEI-2 and MEI-1 along the spindle long axis (Fig. 4 and Fig. S3; and see Materials and methods). In these analyses, signal intensities at a given position along the long axis of a spindle are normalized to the mean intensity for that spindle, enabling evaluation of the distribution of signal in the chromosome-occupied central portion of the spindle relative to the pole regions. Composite plots of line profiles confirmed the enrichment of MEI-2 and MEI-1 near spindle poles for all three genotypes (Fig. 4 C and Fig. S3 C). Moreover, our analysis allowed us to conclude that normalized MEI-2 and MEI-1 signals near chromosomes in the central region of *TBB-1-only* spindles were significantly reduced compared with both *TBB-2-only* and wild-type spindles (Fig. 4 D and Fig. S3 D). Interestingly, MEI-2 localization profiles in *tbb-2(E439K)* spindles were not distinguishable from wild-type controls (Fig. 4, C and D), consistent with previous reports suggesting that the TBB-2(E439K) amino acid change impairs microtubule-katanin interactions without disrupting katanin recruitment (Lu et al., 2004).

These findings regarding katanin localization help to provide a potential mechanistic explanation for the observed differences in sensitivity of spindle structure to partial loss of KLP-18 function: By recruiting less katanin to the spindle midzone relative to the poles, TBB-1-only microtubules may create a region of reduced severing activity, potentially leading to longer antiparallel overlaps in the mid-spindle that preserve spindle stability when cross-linking is compromised. Conversely, the presence of TBB-2 in microtubules may promote severing in two ways, both by helping to recruit katanin to the spindle center and by increasing susceptibility to katanin-mediated severing. We suggest that mixed-isotype microtubules may undergo an “appropriate” level of severing, whereas TBB-2 only microtubules may be subject to higher local severing activity that becomes destructive when cross-linking is reduced—presumably by leading to shortened regions of antiparallel microtubule overlap.

### β-tubulin isotype composition differentially affects meiotic spindle length

**Figure 5.**
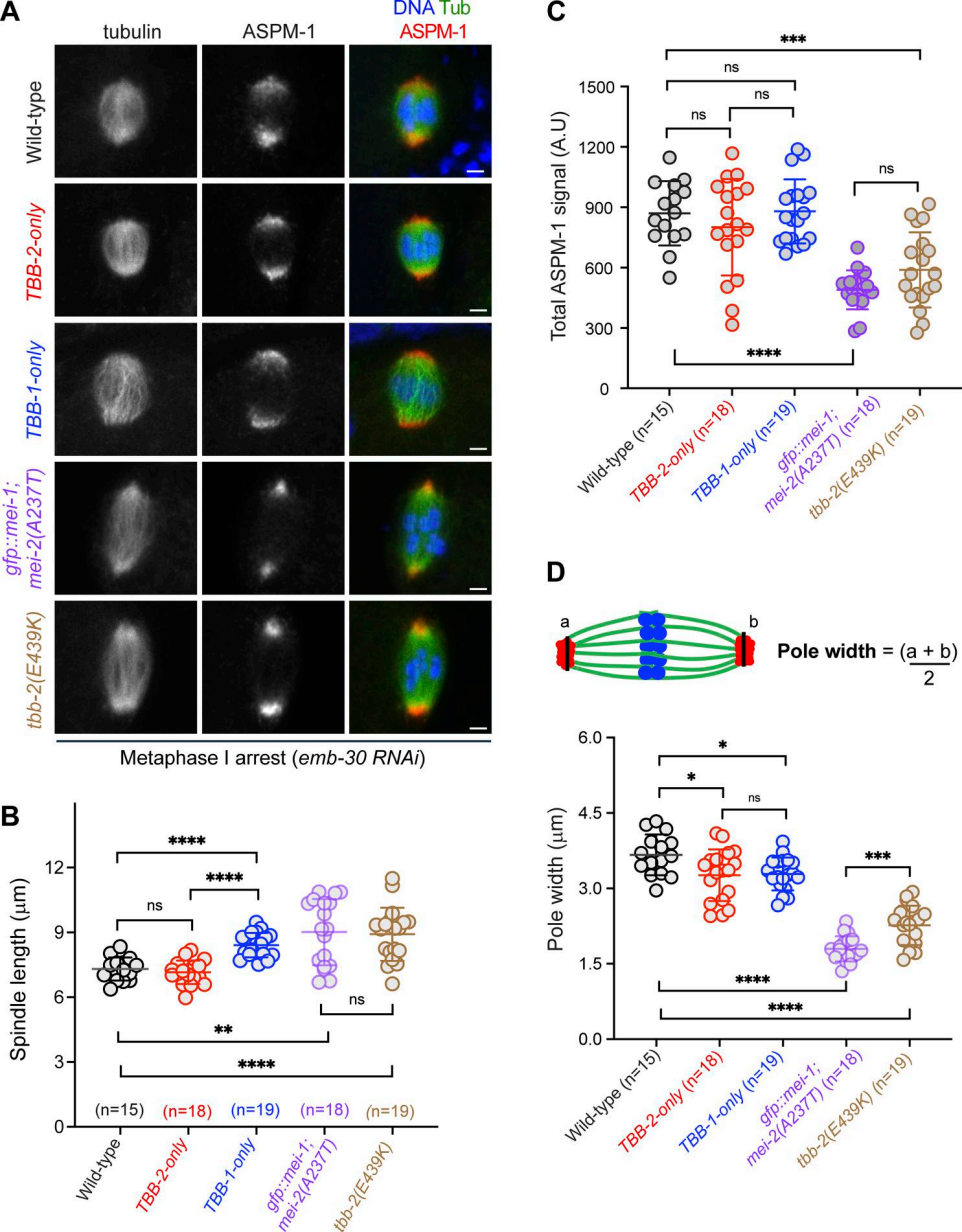
**β-tubulin isotype composition differentially affects meiotic spindle morphology. (A)** Representative IF images of metaphase I-arrested spindles illustrating length differences for the indicated genotypes; scale bar = 2.0 μm. (The *tbb-2(E439K)* strain is wild type at the *tbb-1* locus). **(B)** Quantification of spindle length measurements. **(C)** Quantification of ASPM-1 pole-associated signal. **(D)** Quantification of pole widths, with schematic depicting how pole widths were measured. For Fig. 5, B–D, *n* = number of spindles analyzed, error bars indicate SD. Data were analyzed using two-tailed Mann–Whitney test; ns, not significant; *P < 0.05; **P < 0.01; ***P < 0.001; ****P < 0.0001. IF, immunofluorescence.

The spatial distribution of katanin predicted that TBB-1-only and TBB-2-only spindles might exhibit different structural properties. To test this possibility, we evaluated several aspects of spindle organization in high-resolution immunofluorescence images of α-tubulin and spindle pole marker ASPM-1 in metaphase I–arrested oocyte spindles in a wild-type [*klp-18(+)*] background. We first assessed spindle length, finding that TBB-1-only spindles were significantly longer than both wild-type and TBB-2-only spindles: 8.41 ± 0.56 μm versus 7.30 ± 0.53 μm (wild type, P < 0.0001) and 7.20 ± 0.54 μm (TBB-2-only, P < 0.0001) (Fig. 5, A and B). Notably, mutants with partially reduced katanin activity [*gfp::mei-1; mei-2(A237T)]* or reduced katanin sensitivity [*tbb-2(E439K)]* similarly exhibited longer meiotic spindles, consistent with the possibility that reduced katanin activity/susceptibility may at least partially underlie the *TBB-1-only* spindle-length phenotype.

We also evaluated spindle pole organization, assessing both pole width and total ASPM-1 signal associated with poles, features that are known to be dependent on functional katanin activity (Danlasky et al., 2020; Connolly et al., 2014; and Fig. 5). Comparisons of wild-type, TBB-1-only, and TBB-2-only spindles did not reveal significant differences in total ASPM-1 signal, although both TBB-1-only and TBB-2-only spindles had slightly narrower poles than wild type. These modest differences contrasted sharply with pronounced defects in spindle pole organization detected in the *gfp::mei-1; mei-2(A237T)* and *tbb-2(E439K)* mutants, which displayed diminished ASPM-1 signal and narrower spindle pole structures. These data suggest that although katanin localization is diminished at the midzone in TBB-1-only spindles, katanin activity near the poles is not compromised.

**Figure S4.**
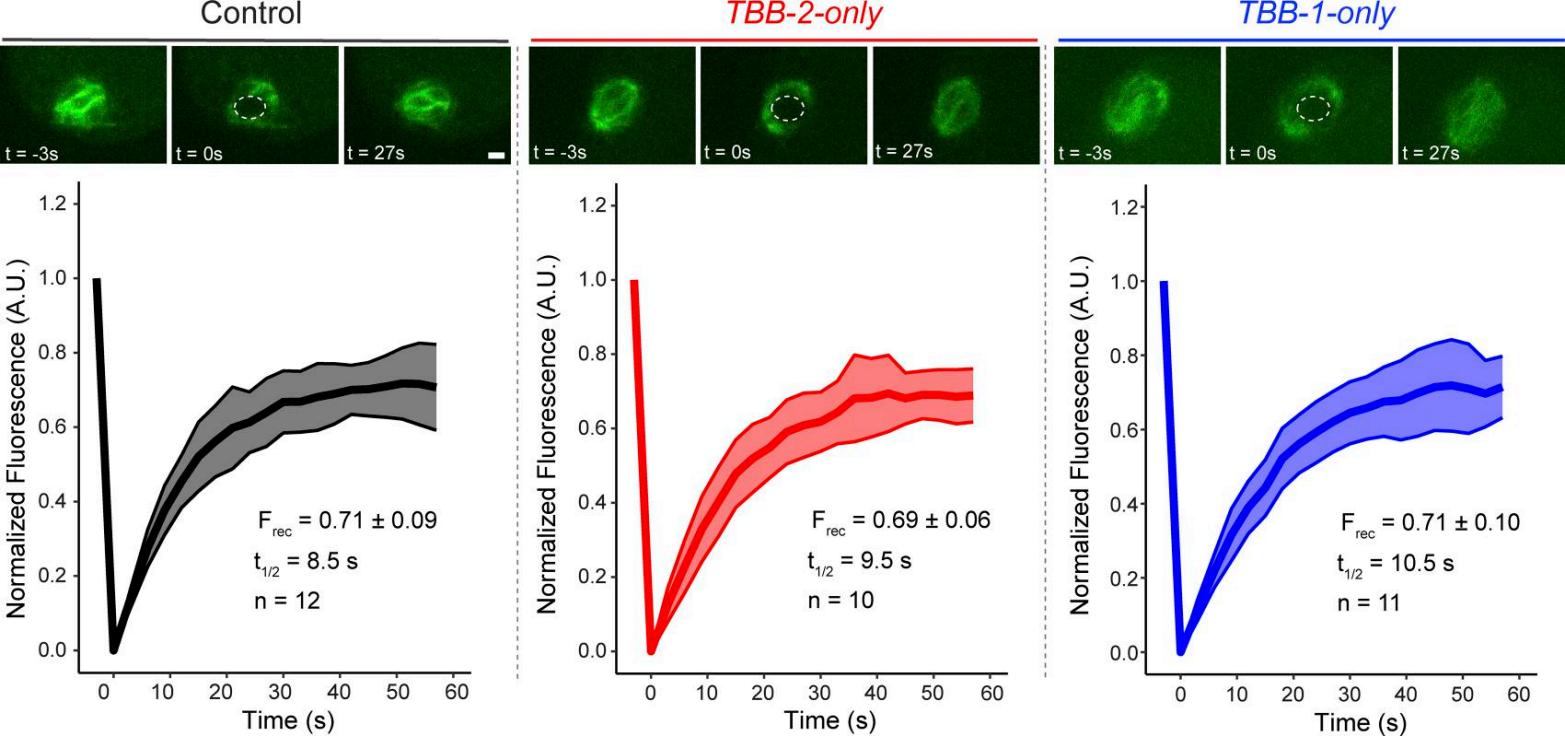
**FRAP analysis did not detect effects of β-tubulin isotype composition on tubulin turnover in oocyte meiotic spindles.** FRAP analysis of GFP-tagged α-tubulin (GFP::TBA-2) was performed on unarrested metaphase I oocyte spindles of the indicated genotypes. (Top) Still images of oocyte spindles from FRAP movies prior to, at the time of, and after photobleaching at the spindle center (white dotted oval). Scale bar = 2.0 μm. (Bottom) Graphs showing the recovery of fluorescence in the bleached region over time. Mean values are indicated by solid lines, and the shaded region corresponds to the SD. The fraction of recovery for each genotype (F_rec_) corresponds to the mean of the plateau values for the individual spindles; plateau values correspond to the mean values for the last five time points. Two-tailed Mann–Whitney tests indicate no significant differences in mean plateau values between the genotypes analyzed. t_½_ values correspond to the x-axis value where the y-axis value corresponds to F_rec_/2.

As it was recently reported that elongation of *C. elegans* oocyte spindles by depletion of the protein kinase ZYG-8 was associated with substantially decreased microtubule turnover (Czajkowski et al., 2024), we also conducted FRAP experiments to assess whether turnover of GFP::tubulin in oocyte metaphase spindles might be affected by β-tubulin isotype composition (Fig. S4; and Videos 1, 2, and 3). However, TBB-1-only and TBB-2-only spindles exhibited similar extent and kinetics of recovery in this FRAP analysis, indicating that effects on tubulin turnover are not sufficient to account for the observed difference in spindle length.

**Video 1.** **FRAP of midzone in unarrested control oocyte spindle.** Time-lapse images of a control metaphase spindle; corresponds to Fig. S4. GFP::tubulin is shown in green. Oocytes were filmed *in utero.* Three frames were acquired prior to bleaching. The rate and extent of recovery was consistent in all movies (*n* = 12). Images were acquired every second, and time elapsed is shown in min:sec. Scale bar = 5.0 μm.

**Video 2.** **FRAP of midzone in unarrested TBB-2-only oocyte spindle.** Time-lapse images of a TBB-2-only metaphase spindle; corresponds to Fig. S4. GFP::tubulin is shown in green. Oocytes were filmed *in utero.* Three frames were acquired prior to bleaching. The rate and extent of recovery was consistent in all movies (*n* = 12). Images were acquired every second, and time elapsed is shown in min:sec. Scale bar = 5.0 μm.

**Video 3.** **FRAP of midzone in unarrested TBB-1-only oocyte spindle.** Time-lapse images of a TBB-1-only metaphase spindle; corresponds to Fig. S4. GFP::tubulin is shown in green. Oocytes were filmed *in utero.* Three frames were acquired prior to bleaching. The rate and extent of recovery was consistent in all movies (*n* = 12). Images were acquired every second, and time elapsed is shown in min:sec. Scale bar = 5.0 μm.

### β-tubulin isotype composition differentially affects properties of microtubule bundles

**Figure 6.**
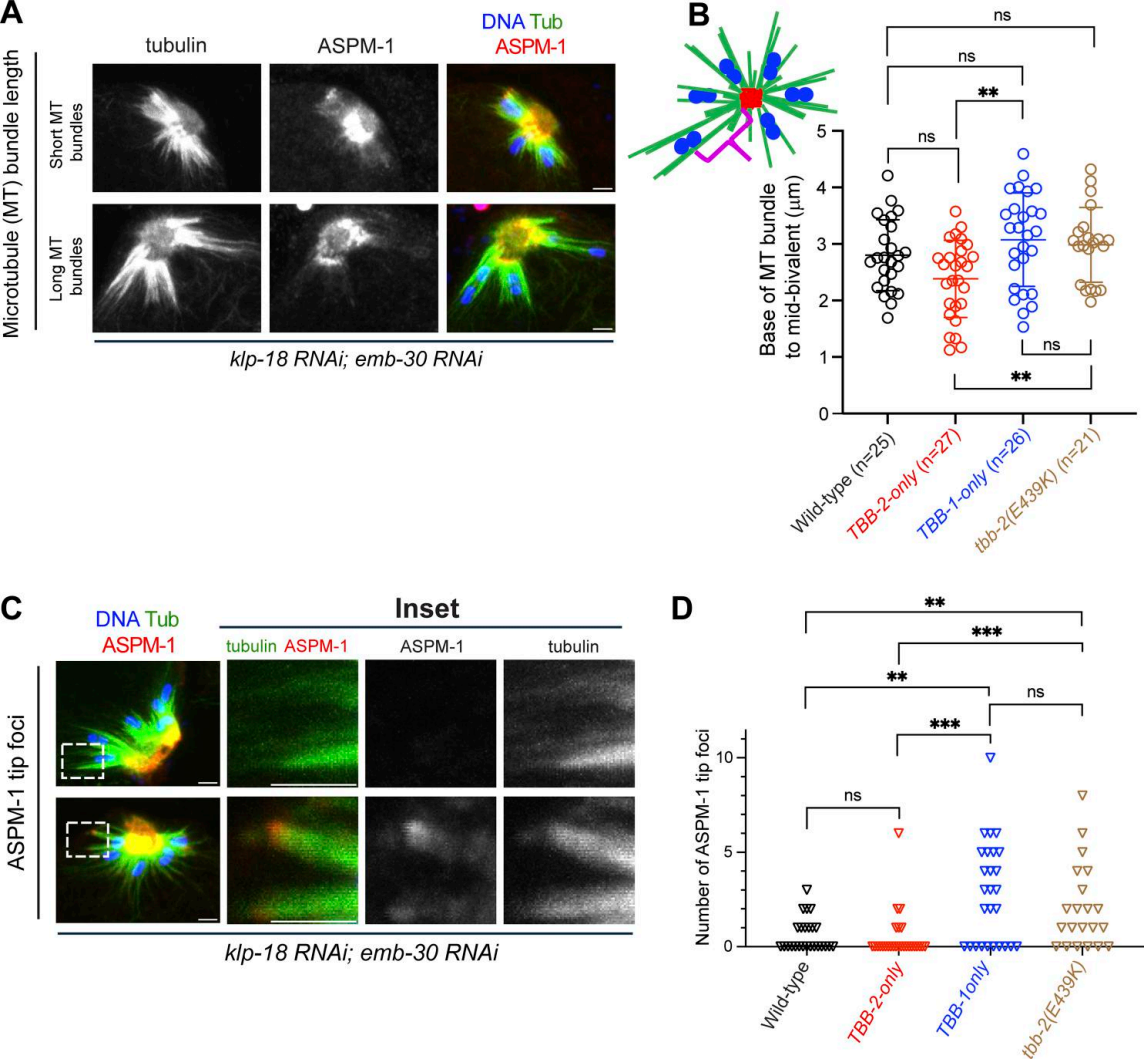
**β-tubulin isotype composition differentially affects properties of microtubule bundles. (A)** IF images of metaphase I–arrested monopolar spindles (*klp-18 RNAi*; *emb-30 RNAi*) showing bivalent positions that reflect microtubule bundle lengths. Scale bars = 2 μm. **(B)** Quantification of distances from images of monopolar spindles of the types depicted in A, measured from the base of a microtubule bundle to the bivalent midpoint, as shown in the schematic diagram (see Results). Each data point represents the mean of the individual base-to-mid-bivalent measurements from a given spindle; error bars indicate SD. **(C)** IF images of monopolar spindles showing presence or absence of ASPM-1 tip foci (insets), which are indicative of antiparallel microtubule organization within bundles despite depletion of KLP-18. Scale bar = 2 μm (whole-spindle images); 5 μm (insets). **(D)** Quantification of the number of ASPM-1 tip foci observed at the periphery of each monopolar spindle. Each data point represents an individual monopolar spindle. Data were analyzed using two-tailed Mann–Whitney tests; ns, not significant; **P < 0.01; ***P < 0.001. IF, immunofluorescence.

We used monopolar spindles generated by depletion of KLP-18 by RNAi to seek additional evidence for differential microtubule properties affected by β-tubulin isotypes (Fig. 6). In this monopolar spindle configuration, lateral associations with microtubules align chromosome bivalents roughly parallel to groups of microtubule bundles that emanate from a region of high microtubule density in the area of the monopole (marked by high ASPM-1 signal) (Wignall and Villeneuve, 2009). In this context, bivalents are transported away from the monopole toward the bundle tips via chromokinesin activity concentrated at the mid-bivalent (Wignall and Villeneuve, 2009). This “ends out” organization creates an opportunity to evaluate potential effects of β-tubulin isotype composition on the relative lengths of microtubule bundles. However, because bundles taper and become fainter toward their tips, it is difficult to make definitive calls regarding where a microtubule bundle ends. Thus, rather than measuring bundle lengths directly, we instead measured the distance from the base of the microtubule bundles to the mid-bivalent, which is a reliably quantifiable measurement that is related to (albeit shorter than) the lengths of the microtubule bundles on which the bivalents are transported (see Material and methods). TBB-1-only monopolar spindles did indeed exhibit significantly longer base-to-mid-bivalent distances compared with TBB-2-only monopolar spindles (3.47 ± 0.95 μm versus 2.78 ± 0.82 μm; P < 0.01) (Fig. 6, A and B), suggesting longer microtubule bundles and consistent with the longer spindle lengths measured for TBB-1-only bipolar spindles. Base-to-mid-bivalent distances in *tbb-2(E439K)* monopolar spindles were also significantly longer than in *TBB-2-only* (P = 0.0091), consistent with the longer spindle lengths observed when microtubule susceptibility to katanin activity is reduced.

Further, closer scrutiny of ASPM-1 localization in these monopolar spindles revealed an additional phenotype. While the vast majority of ASPM-1 is localized at the monopoles, i.e., at the bases of the microtubule bundles, ASPM-1 foci are sometimes detected at the periphery of these monopolar spindles, at the tips of the microtubule bundles distal to the monopole (Czajkowski et al., 2024). *TBB-1-only* and *tbb-2(E439K)* monopolar spindles had a higher incidence of microtubule bundles with ASPM-1 foci at their peripheral tips than was observed in both the wild-type and *TBB-2-only* genetic backgrounds (Fig. 6, C and D). As ASPM-1 is considered to be a marker of microtubule minus ends (Wignall and Villeneuve, 2009; Czajkowski et al., 2024), ASPM-1 foci at the peripheral tips suggests the presence of antiparallel microtubule organization within such bundles.

### Live imaging reveals hidden contributions of β-tubulin isotype composition to spindle morphology and chromosome separation dynamics

**Figure 7.**
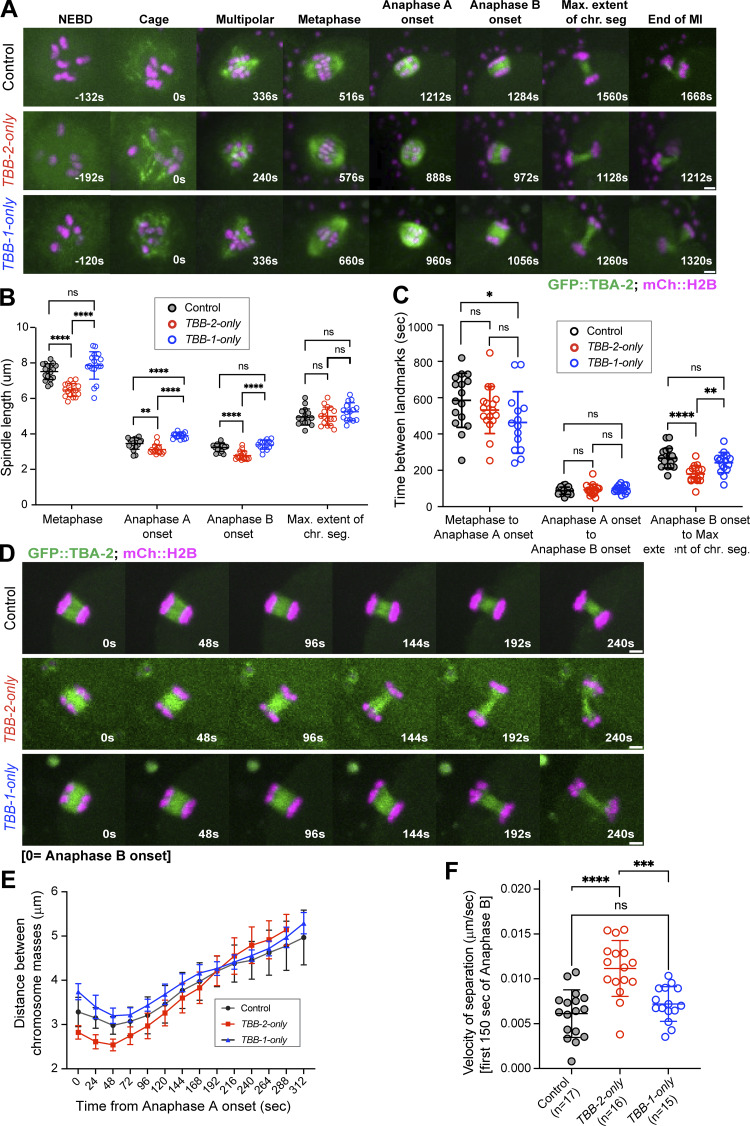
**Live imaging reveals contributions of β-tubulin isotype composition to spindle morphology and chromosome separation dynamics. (A)** Representative maximum intensity projection images from time-lapse movies of live oocytes expressing GFP::TBA-2 to mark microtubules and mCherry::H2B to mark chromosomes, showing meiosis I landmarks. For our analysis, t = 0 is the onset of meiotic spindle assembly as defined by the appearance of microtubules surrounding the chromosomes (cage stage). Scale bar = 2.0 μm. **(B)** Quantification of spindle length at specified meiotic landmarks; error bars indicate SD. **(C)** Timing of meiosis I spindle assembly measurements between specified meiotic landmarks; error bars indicate SD. **(D)** Representative maximum intensity projection images from time-lapse movies of anaphase B. Anaphase B onset (t = 0) is defined as the point when the spindle has achieved its shortest length and the separating chromosome masses have reached opposite poles of the spindle. **(E)** Quantification of chromosome separation distances (mean ± SD) over time during anaphase I. **(F)** Quantification of anaphase B chromosome separation velocity during meiosis I for the first 150 s after anaphase B onset (see Materials and methods); *n* = number of spindles analyzed. Error bars indicate SD. For Fig. 7, B, C, and F, each data point represents a spindle, and data were analyzed using two-tailed Mann–Whitney test; ns, not significant; *P < 0.05; **P < 0.01; ***P < 0.001; ****P < 0.0001.

**Figure S5.**
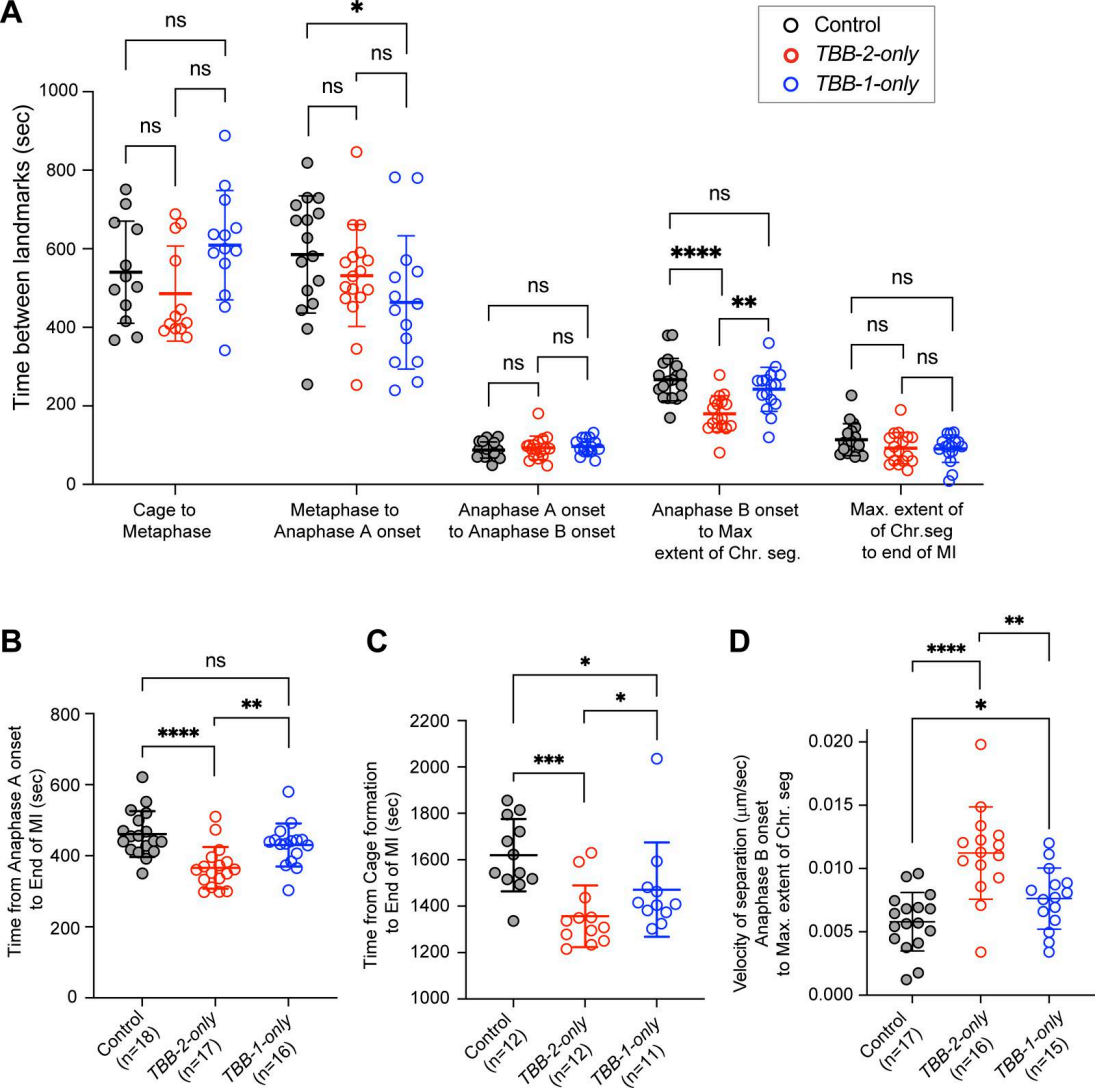
**Additional analysis for live imaging presented in Fig. 7. (A–C)** As in Fig. 7 C, with additional landmark-to-landmark intervals included. **(D)** As in Fig. 7 F, with a different endpoint. ns, not significant; *P < 0.05; **P < 0.01; ***P < 0.001; ****P < 0.0001.

To investigate β-tubulin isotype contributions to the dynamics of meiotic spindle assembly and function, we used spinning disk confocal microscopy to conduct time-lapse *in utero* imaging of control, *TBB-1-only*, and *TBB-2-only* oocytes expressing GFP-tagged α-tubulin (GFP::TBA-2) and mCherry::H2B, to mark microtubules and chromosomes, respectively (Fig. 7; and Videos 4, 5, and 6). These experiments build on pioneering liveimaging studies of *C. elegans* oocyte meiosis (McNally et al., 2006; Chuang et al., 2020) that defined landmark events and provide a framework for dissecting the roles of individual molecular components in both spindle formation and chromosome segregation dynamics. For our analyses of progression through meiosis I, we designated the appearance of microtubules surrounding the chromosomes (“Cage” stage) as the onset of meiotic spindle assembly (t = 0), and we used the timing of metaphase, anaphase A onset, anaphase B onset, and maximum extent of chromosome segregation as landmarks to align and compare morphology and timing for control, TBB-1-only, and TBB-2-only spindles (Fig. 7 A and Fig. S5; and Materials and methods). Several interesting observations emerged from these analyses:

**Video 4.** **Time-lapse images of control oocyte expressing GFP::TBA-2 to mark microtubules and mCherry::H2B to mark chromosomes; corresponds to Fig. 7 and Fig. S5.** Images were captured at 12-s intervals and represent composite maximum projections of z-stacks spaced 2.0 μm apart. Scale bar = 5.0 μm.

**Video 5.** **Time-lapse images of TBB-2-only oocyte expressing GFP::TBA-2 to mark microtubules and mCherry::H2B to mark chromosomes; corresponds to Fig. 7 and Fig. S5.** Images were captured at 12-s intervals and represent composite maximum projections of z-stacks spaced 2.0 μm apart. Scale bar = 5.0 μm.

**Video 6.** **Time-lapse images of TBB-1-only oocyte expressing GFP::TBA-2 to mark microtubules and mCherry::H2B to mark chromosomes; corresponds to Fig. 7 and Fig. S5.** Images were captured at 12-s intervals and represent composite maximum projections of z-stacks spaced 2.0 μm apart. Scale bar = 5.0 μm.

### TBB-2-only metaphase spindles are short but assemble with normal timing

In these liveimaging experiments, TBB-2-only metaphase spindles were significantly shorter than both control spindles (6.47 ± 0.36 μm versus 7.51 ± 0.45 μm; P < 0.0001) and TBB-1-only spindles (7.86 ± 0.77 μm; P < 0.0001) (Fig. 7 B). We note that this finding is only partially consistent with our measurements of spindle lengths in immunofluorescence imaging of metaphase I–arrested oocyte spindles (Fig. 5), where we detected a length difference between TBB-2-only spindles and TBB-1-only spindles, but not between TBB-2-only spindles and those from wild-type controls; this apparent discrepancy is likely caused by the prolonged arrest in the Fig. 5 experiments, as discussed below. Despite differences in metaphase spindle lengths, we did not detect differences in the time to assemble bipolar metaphase spindles (Fig. S5 A).

### Despite assembling shorter spindles, TBB-2-only oocytes achieved a normal extent of chromosome separation during anaphase

The lengths of TBB-2-only spindles were significantly shorter than control and TBB-1-only spindles not only at metaphase, but also at both anaphase A onset and anaphase B onset. Nevertheless, TBB-2-only spindles ultimately achieved maximum chromosome separation distances comparable with those of the other genotypes (∼5.0 μm in all cases) (Fig. 7 B).

### TBB-2-only-microtubules promote accelerated spindle elongation and increased chromosome separation velocity during anaphase B

TBB-2-only spindles reached maximum anaphase B chromosome separation significantly faster than control and TBB-1-only spindles: 180 ± 46 s from anaphase B onset versus 267 ± 54 s in control (P < 0.0001) and versus 242 ± 56 s in TBB-1-only oocytes (P = 0.0012) (Fig. 7 C and Fig. S5 A). This reflects an elevated rate of chromosome separation during anaphase B in TBB-2-only oocytes, as revealed by monitoring of chromosome separation distances throughout anaphase B (Fig. 7 D) and visualized by plotting composite measurements of distance vs. time (Fig. 7 E) or by plotting average separation velocity measurements for individual spindles (Fig. 7 F and Fig. S5 D).

### TBB-1-only spindles exhibit increased susceptibility to structural failure during prolonged arrest

**Figure 8.**
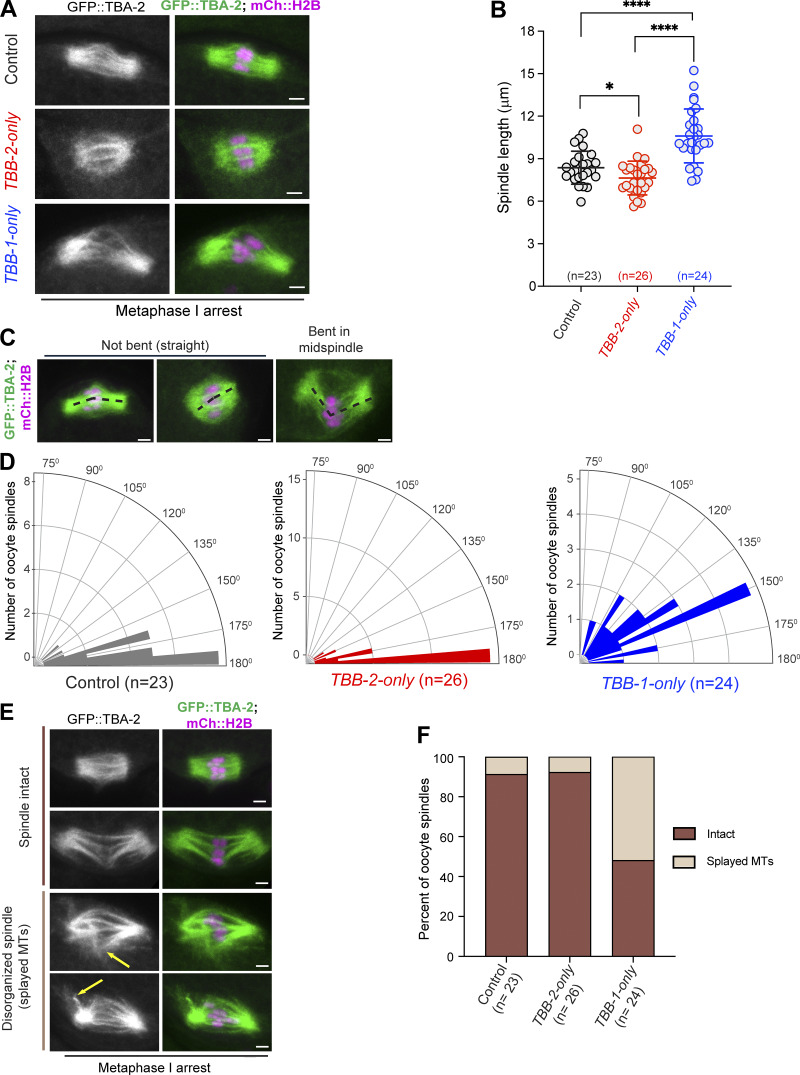
**TBB-1-only spindles exhibit increased susceptibility to structural failure during prolonged arrest. (A)** Representative fixed images of metaphase I–arrested spindles in control, TBB-2-only, and TBB-1-only oocytes expressing GFP::TBA-2 and mCherry::H2B. **(B)** Quantification of meiotic spindle length in A; TBB-1-only spindles are significantly longer than wild-type and TBB-2-only spindles. Error bars indicate SD. Data were analyzed using two-tailed Mann-Whitney test; *P < 0.05; ****P < 0.0001. **(C)** GFP::TBA-2 and mCherry::H2B fixed fluorescence images illustrating bent midspindle morphology. **(D)** Quantification of spindle angle as illustrated in C. TBB-1-only spindles are significantly bent (P < 0.0001 vs control, and P < 0.0001 vs TBB-2-only; two-tailed Mann–Whitney test), reflecting structural failure. **(E)** GFP::TBA-2 and mCherry::H2B fixed fluorescence images illustrating splaying of microtubules from mid-spindle and pole regions. **(F)** Quantification of microtubule splaying phenotype illustrated in E. Scale bars in all images = 2.0 μm.

We set out to investigate the basis of differences between our live imaging of oocyte meiosis and immunofluorescence imaging of metaphase-arrested spindles regarding effects of β-tubulin isotype composition on metaphase spindle length. To this end, we imaged GFP::TBA-2 and mCherry::H2B fluorescence in fixed metaphase I–arrested oocytes from the strains we had used for live imaging. This analysis largely recapitulated our previous finding that metaphase-arrested TBB-1-only spindles are significantly longer than both wild-type and TBB-2-only spindles (Fig. 8, A and B), supporting the interpretation that prolonged arrest may exacerbate the effects of a modest functional difference between mixed-isotype and TBB-1-only microtubules. Notably, we also observed additional structural abnormalities (e.g., fractured or bent spindle or microtubules splaying from mid-spindle or pole regions) in metaphase-arrested TBB-1-only spindles in the *GFP::TBA-2; mCherry::H2B* genetic background (Fig. 8, C–F). As such abnormalities were not detected in immunofluorescence experiments using untagged strains, we infer that GFP::TBA-2 may contribute to defects revealed in the context of meiotic arrest. The presence of GFP::TBA-2 may likewise be the reason that these experiments detected a small but significant reduction in length of metaphase-arrested TBB-2 only spindles (relative to wild type) that was not detected in untagged strains.

## Discussion

**Figure 9.**
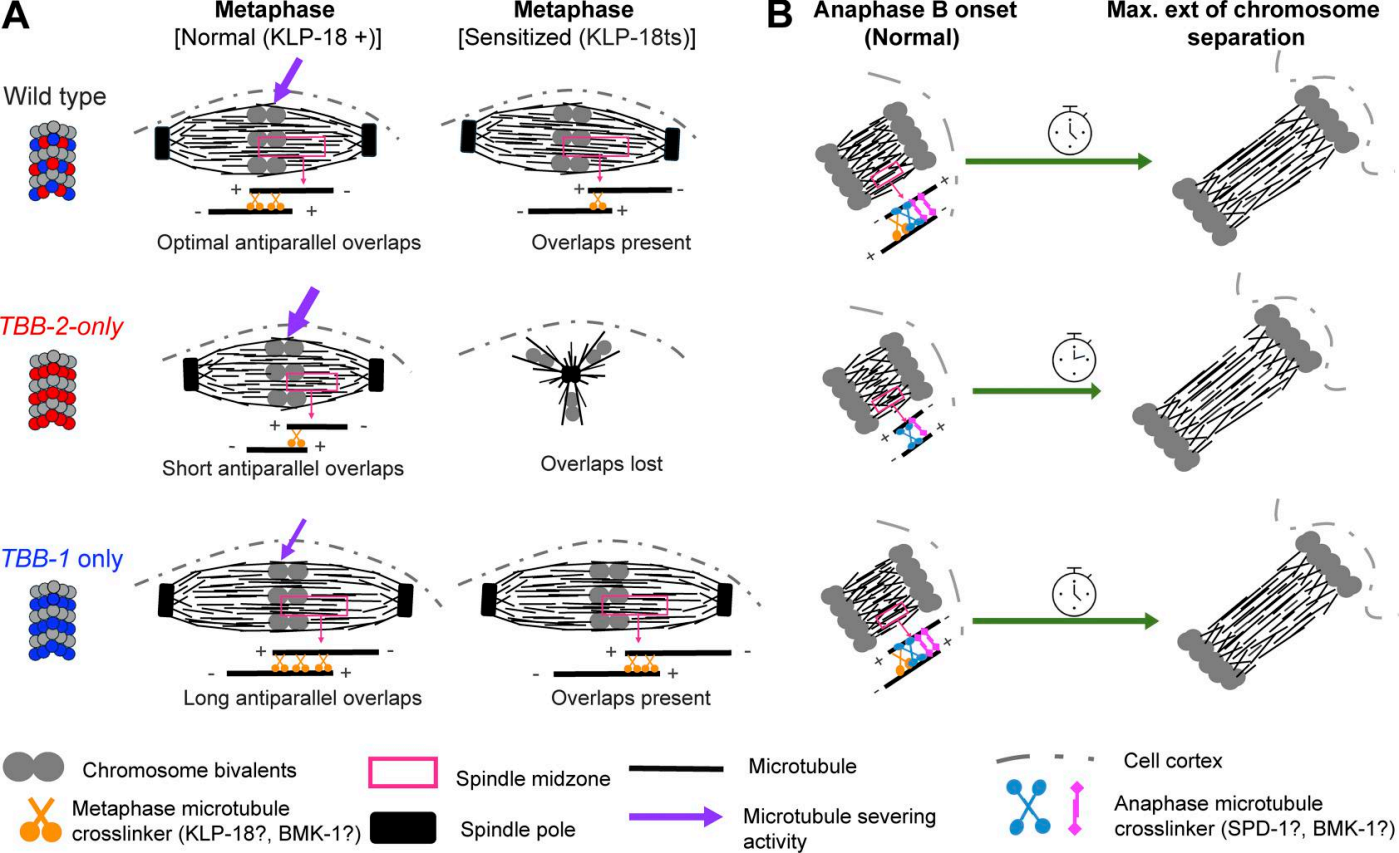
**Model depicting inferred contributions of β-tubulin isotype composition to bipolar spindle assembly and anaphase chromosome separation. (A)** Diagram depicting a model in which assembly of bipolar oocyte spindles requires a balance between microtubule crosslinking and severing activities, and β-tubulin isotype composition of microtubules helps to maintain this balance. Collectively, our data suggest that differential localization of katanin and/or differential sensitivity to katanin-mediated severing, conferred by β-tubulin isotype composition, affects microtubule length. Accordingly, microtubules would be shorter when only TBB-2 is present, leading to shorter microtubule overlaps; conversely, microtubules would be longer when only TBB-1 is present, leading lead to longer overlaps. When cross-linking motors are not perturbed (KLP-18+), the combined effects of these changes can affect the length of bipolar spindles. In the sensitized context when KLP-18 microtubule crosslinking activity is compromised (KLP-18ts), the residual cross-linking activity is sufficient to mediate antiparallel overlaps that can maintain spindle bipolarity when the microtubules are long enough (i.e., when they contain TBB-1); however, the residual cross-linking activity cannot support sufficient antiparallel overlap between shorter (TBB-2-only) microtubules, resulting in monopolar spindle organization. We propose that the presence of both TBB-1 and TBB-2 isotypes in wild-type oocytes results in an optimal balance between microtubule severing and crosslinking activities. **(B)** Diagram depicting proposed effects of β-tubulin isotype composition on anaphase B spindle elongation and chromosome separation dynamics. We speculate that during anaphase B, shorter microtubules in TBB-2-only spindles leads to shorter antiparallel overlaps and reduced cross-linking (e.g., by anaphase cross-linkers such as BMK-1 and SPD-1 [Li et al., 2023]), making these spindles less resistant to activities of sliding motors and thereby enabling rapid spindle elongation. In contrast, we speculate that microtubule crosslinking is robust in both TBB-1-only and mixed-isotype spindles due to longer antiparallel overlaps, making these spindles more resistant to sliding and hence slowing spindle elongation.

The research reported here was initiated with the goal of understanding how different β-tubulin isotypes contribute to assembly and function of oocyte meiotic spindles, which form and segregate chromosomes in the absence of centrosomes. Our first evidence regarding differential contributions of the TBB-1 and TBB-2 isotypes during oocyte meiosis came from experiments in which the microtubule-crosslinking motor KLP-18 was compromised, which revealed a spindle assembly vulnerability in worms expressing only the TBB-2 isotype that could be suppressed by mutations that reduce katanin susceptibility or activity. These findings led us to investigate the effects of β-tubulin isotype composition on the morphology and behavior of meiotic spindles under conditions where cross-linking motors were not perturbed. Collectively, our data are consistent with a model in which β-tubulin isotype composition helps to maintain a balance between microtubule-crosslinking and severing activities during oocyte meiosis to reliably establish spindle bipolarity, maintain spindle structural integrity during stress, and achieve normal dynamics of chromosome separation (Fig. 9).

Our findings regarding spindle bipolarity and metaphase spindle dimensions can be explained by the model depicted in Fig. 9 A. We propose that differential localization of katanin and/or differential susceptibility to katanin-mediated severing, properties conferred by β-tubulin isotype composition, affect the lengths of microtubules in assembling meiotic spindles. Accordingly, microtubules would be shorter when only the TBB-2 isotype is present, which would in turn result in shorter regions of overlap between crosslinked microtubules; conversely, microtubules would be longer when only TBB-1 is present, leading lead to longer overlaps. When crosslinking motors are not perturbed, the combined effects of isotype composition on the lengths of microtubules and the lengths of crosslinked overlaps can affect the length of bipolar spindles. In the sensitized context when KLP-18 microtubule-crosslinking activity is compromised, spindle assembly becomes vulnerable to modulation of microtubule length. We propose that when the microtubules are long enough (i.e., when they contain TBB-1), the residual crosslinking activity is sufficient to mediate antiparallel overlaps that can support spindle bipolarity. However, when microtubules are too short (TBB-2-only), compromised crosslinking activity can lead to loss of antiparallel overlaps, resulting in monopolar spindle organization.

While TBB-1-only microtubules perform better than TBB-2-only microtubules in assembling bipolar meiotic spindles when KLP-18–mediated crosslinking is compromised, our data indicate that there is nevertheless a potential downside to lacking the TBB-2 isoform. Specifically, we found that the longer meiotic spindles assembled from TBB-1-only microtubules exhibited an increased incidence of structural failure in the context of metaphase arrest. This suggests that increased antiparallel microtubule overlap, while helping to ensure spindle bipolarity, may ultimately render spindles vulnerable to increased outward sliding forces. We infer that mixed-isotype microtubules may help to achieve an optimal balance between the microtubules and the proteins that mediate interactions between them.

Our live imaging analysis revealed additional contributions of β-tubulin isotype composition to oocyte meiotic spindle function. Most notably, our examination of anaphase B revealed that TBB-2-only spindles elongate significantly faster and exhibit chromosome separation velocities 67% and 43% faster than control and TBB-1-only spindles, respectively. As depicted in Fig. 9 B, we speculate that shorter microtubules may also underlie this faster chromosome separation phenotype of TBB-2-only spindles, perhaps by affording fewer cross-links between antiparallel microtubules and thereby offering reduced resistance to sliding and/or increasing the likelihood that anaphase motors will smoothly slide microtubules apart. Consistent with this interpretation, it was recently shown that combinatorial depletion of microtubule-crosslinking factors BMK-1/kinesin-5 and SPD-1/PRC1 leads to accelerated anaphase B spindle elongation and enhanced chromosome separation velocity (Li et al., 2023). In principle, β-tubulin isotype composition could also potentially affect the rate of spindle elongation through effects on microtubule dynamics; however, we note that our FRAP analysis did not detect any major impact of β-tubulin isotype on tubulin dynamics in metaphase I spindles.

While the current work has clearly demonstrated that the TBB-1 and TBB-2 isotypes make distinct contributions to the properties of acentrosomal spindles during *C. elegans* meiosis, future work will be needed to uncover the molecular determinants underlying the observed functional differences. The carboxy-terminal tail region (CTT) is likely to be involved, as 8 of the 12 amino acid differences between TBB-1 and TBB-2 are in the CTT, including the residue affected by the *tbb-2(E439K)* missense mutation inferred to reduce susceptibility to katanin (Lu et al., 2004). Tubulin CTTs are short intrinsically disordered regions located on the outer surfaces of microtubules, where they serve as major sites for interactions with molecular motors and microtubule-associated proteins and are extensively modified by posttranslational modifications (PTMs) such as glutamylation, glycylation, and tyrosination (reviewed by Roll-Mecak, 2020). Further, β-tubulin CTT peptides are sufficient to stimulate katanin ATPase activity (Zehr et al., 2020), and polyglutamylation of α-tubulin and β-tubulin can modulate katanin severing activity *in vitro* (Szczesna et al., 2022). Thus, we speculate that the amino acid differences between the TBB-1 and TBB-2 isotypes may contribute to differential PTM of the CTTs and/or work in concert with PTMs to achieve differential interactions with motors, crosslinkers, and severing enzymes.

Interestingly, the same balance of tubulin isotypes that supports robustness of acentrosomal spindle assembly and function during oocyte meiosis must later support reliable assembly and function of centrosome-organized mitotic spindles during the early embryonic cell divisions (Müller-Reichert et al., 2010). Honda et al. (2017) also uncovered differential contributions of β-tubulin isotypes to functional properties of mitotic spindles. By using a tagged plus-end–binding protein to investigate the dynamics of astral microtubules in one-cell embryos, they found that TBB-2-only microtubules were more stably elongating in this context, whereas TBB-1-only microtubules were more dynamic. At a first glance, these findings might initially appear at odds with our analysis of differential contributions of β-tubulin isotypes to oocyte meiosis, where TBB-2 is associated with shorter spindles/microtubule bundles and TBB-1 is associated with longer spindles/microtubule bundles. However, as discussed above, these meiotic spindle phenotypes can be explained by isotype differences affecting localization of and sensitivity to katanin, which is highly abundant during meiosis but is actively degraded at the oocyte-to-embryo transition (Pintard et al., 2003; Joly et al., 2020). Thus, the properties exhibited by microtubules of similar composition can differ substantially in the very different cellular environments in which meiotic and mitotic spindles form.

There are two major take-home messages from this work:

First, our studies have provided evidence that oocyte meiosis can be viewed as a balancing act between microtubule severing activities and microtubule crosslinking activities. Our findings support a model in which severing and crosslinking activities must counterbalance each other to form bipolar spindles and segregate chromosomes in the absence of centrosomes and implicate β-tubulin isotype composition in helping to maintain this crucial balance.

Second, our data contribute to a growing appreciation that superficially redundant tubulin isotype variants can make distinct contributions that become relevant under specific cellular conditions. This phenomenon has been previously shown in budding yeast, where α-tubulin isotype substitution strains expressing either Tub1 only or Tub3 only are both viable under normal growth conditions but display differential sensitivity to the microtubule-depolymerizing drug benomyl (Nsamba et al., 2021). Additionally, the two α-tubulin isotypes contribute antagonistic properties required for optimal spindle elongation and length control during mitosis (Nsamba et al., 2025). Likewise in fission yeast, substitution of the major α-tubulin Nda2 with minor isotype Atb2 does not affect vegetative growth, but Atb2-only cells display major defects in sporulation and meiosis (Chen et al., 2025). Further, the *C. elegans* TBB-1 and TBB-2 β-tubulin isotypes have distinct effects on thermal tolerance and sensitivity to the benzimidazole derivative albendazole during larval growth (Pallotto et al., 2022) and on astral microtubule dynamics in the mitotic spindles of one-cell embryos (Honda et al., 2017).Thus, rather than simply representing functionally interchangeable components, tubulin isotypes can confer distinct properties and behaviors to microtubules that ensure robust performance of microtubule-based structures under varying cellular conditions.

## Materials and methods

### *C. elegans* strains

A list of *C. elegans* strains used in this study is provided in Table 1.

**Table 1.** *C. elegans* strains used

Strain	Genotype	Reference
N2	Wild type	Brenner (1974)
VC167	***tbb-2(0)*** *tbb-2(gk130) III*	Wright and Hunter (2003), RRID:WB-STRAIN:WBStrain00035552
VC364	***tbb-1(0)*** *tbb-1(gk207) III*	Wright and Hunter (2003), RRID:WB-STRAIN:WBStrain00035711
SA1057	***TBB-1-only*** *tbb-2(tj41[tbb-2[tbb-1 substitution]]) III*	Honda et al. (2017), RRID:WB-STRAIN:WBStrain00063116
SA898	*tbb-1(tj30[gfp::tbb-1]) unc-119(ed3)III; tjIs72[pie-1promoter-mCherry::H2B pie-1 promoter-2XmCherry::tbg-1 +unc119(+)]*	Honda et al. (2017)
SA899	*tbb-2(tj26[gfp::tbb-2]) unc-119(ed3)III; tjIs72[pie-1promoter-mCherry::H2B pie-1 promoter-2XmCherry::tbg-1 +unc119(+)]*	Honda et al. (2017)
SA1108	***TBB-2-only*** *tbb-1(tj59[tbb-1[tbb-2 substitution]]) III*	Honda et al. (2017), RRID:WB-STRAIN:WBStrain00063118
EU1329	***klp-18ts*** *klp-18(or447ts) IV*	Connolly et al. (2014) (Maintained at 15°C)
AV1259	*tbb-1(gk207) III; klp-18(or447ts) / tmC5 [F36H1.3(tmIs1220)] IV*	This study
AV1294	*tbb-2(tj41[tbb-2[tbb-1 substitution]]) III; klp-18(or447ts) / tmC5 [F36H1.3(tmIs1220)] IV*	This study
AV1295	*tbb-1(tj59[tbb-1[tbb-2 substitution]]) III; klp-18(or447ts) / tmC5 [F36H1.3(tmIs1220)] IV*	This study
AV1301	*tbb-2(gk130) III; klp-18(or447) / tmC5[F36H1.3(tmIs1220)] IV*	This study
AV1314	*tbb-1(tj59[tbb-1[tbb-2 substitution]]), tbb-2(me196 E439K) III; klp-18(or447ts) / tmC5 [F36H1.3(tmIs1220)] IV*	This study
AV1340	*mei-2(me198 A237T) I; tbb-1(tj59[tbb-1[tbb-2 substitution]]) III; klp-18(or447ts) / tmC5 [F36H1.3(tmIs1220)] IV*	This study
AV1342	*mei-2(me198 A237T), mei-1(wow21[ZF:GFP:mei-1]) I; zif-1(gk117) III*	This study
AV1348	*tbb-2(me196 E439K) III* [*me196* is re-creation of *tbb-2(sb26)*]	Lu et al. (2004)
AV1393	*mei-2(me198, A237T) I* [*me198* is re-creation of *mei-2(ct98)*]	Srayko et al. (2000)
OD57	*unc-119(ed3) III;; ltIs25[pAZ132; pie-1::GFP::tba-2; unc-119 (+)] IV; ltIs37 [pAA64; pie-1/mCherry::his-58; unc-119 (+)]*	Dumont et al. (2010)
AV1361	*tbb-2(tj41[tbb-2[tbb-1 substitution]]) III; ltIs25[pAZ132; pie-1::GFP::tba-2; unc-119 (+)] IV; ltIs37 [pAA64; pie-1/mCherry::his-58; unc-119 (+)]*	This study
AV1362	*tbb-1(tj59[tbb-1[tbb-2 substitution]]) III; ltIs25[pAZ132; pie-1::GFP::tba-2; unc-119 (+)] IV; ltIs37 [pAA64; pie-1/mCherry::his-58; unc-119 (+)]*	This study

Italics are used for all components of gentotypes in standard C. elegans nomenclature.

Bold elements for some genotypes are used to highlight abbreviated genotype nicknames used in text and figures, and are explained therein.

Except where noted, worms were cultured under standard conditions (Stiernagle, 2006) at 20°C. For experiments involving *tbb-1(0); klp-18ts* or *TBB-2-only; klp-18ts* worms, homozygous mutant worms were derived from strains carrying *klp-18ts* in *trans* to the *tmC5* balancer. Bold elements for some strains are used to highlight abbreviated genotype nicknames used in text and figures.

### RNAi feeding

RNAi was done as previously described (Wolff et al., 2022). Briefly, individual RNAi clones from an RNAi feeding library (Kamath et al., 2003) were streaked on LB plates containing ampicillin and grown overnight at 37°C. The next day, single colonies were grown overnight at 37°C in LB with 100 μg/ml ampicillin for 16–18 h. Overnight cultures were spun down, resuspended, and plated on nematode growth medium (NGM) plates containing 100 μg/ml ampicillin and 1 mM IPTG. Plates were dried in a dark space overnight at 23°C. On the same day, worms were synchronized by bleaching of gravid adults, collecting their embryos, and plating embryos on unseeded plates to hatch overnight at 20°C. The next day, newly hatched L1 worms were transferred to appropriate RNAi plates and grown to adulthood at 20°C for 3–4 days.

### Immunofluorescence and antibodies

Immunofluorescence was performed as described by Wolff et al. (2022). Adult gravid worms were picked into a 5 μl drop of sterile M9 (22 mM KH2PO4, 42 mM Na2HPO4, 85.5 mM NaCl, and 1 mM MgSO4) on glass coverslips (22 × 22 mm) and were dissected to release embryos. A poly-L-lysine slide was laid on top to pick up the coverslip and then plunged into liquid nitrogen for 5 min. The coverslip was rapidly flicked off via razor blade, and slides were fixed in cold methanol for 35 min at −20°C. Slides were rehydrated twice in 1X PBS with 0.1% Triton-X-100 (PBS-T) and then blocked in AbDil buffer (1X PBS with 4% BSA, 0.1% Triton-X-100, and 0.02% sodium azide) at room temperature for at least 60 min. Primary antibodies were diluted in AbDil, and slides were incubated in a humid chamber overnight at 4°C. The following day, slides were washed three times with PBS-T and incubated in secondary antibodies diluted in AbDil for 2 h (in the humid chamber) at room temperature. Samples were rinsed again three times in PBS-T as before and incubated in Hoechst (D1306; Invitrogen at 1:1,000 in PBS-T) for 12–15 min at room temperature. The samples were washed one final time twice in PBS-T and mounted in 5–6 μl of mounting media (0.5% p-phenylenediamine, 20 mM Tris-Cl, pH 8.8, and 90% glycerol), and a coverslip (22 × 22 mm #1) was gently laid on top and then sealed with nail polish. Mounted slides were stored at 4°C.

Primary antibodies used in this study were rabbit-α-ASPM-1 (1:5,000, [Wignall and Villeneuve, 2009]), rabbit-α-MEI-1 (1:100, gift from Frank McNally, University of California, Davis, CA, USA), rabbit-α-MEI-2 (1:100, [McNally et al., 2014]), rabbit-α-KLP-18 (1:10, 000, gift from Olaf Bossinger, University of Cologne, Cologne, Germany) and directly conjugated mouse-α-Tubulin-FITC, RRID:AB_476967 (1:500, DM1α, Sigma-Aldrich). Alexa Fluor–conjugated secondary antibodies (Invitrogen) were used at 1:500. Fixed imaging was performed at 4°C on an Inverted Zeiss LSM 880 Laser Scanning Confocal Microscope with a 63× (Plan APO for AiryScan) oil (NA = 1.58) objective. The microscope is equipped with a Piezo-driven motorized stage (PZ-2000 XYZ, Applied Scientific Instrumentation). Z-stacks were obtained at 0.3-μm increments. Undeconvolved images were processed with ImageJ, RRID:SCR_003070. Unless otherwise stated, images in the paper are shown as maximum intensity projections. For analysis of spindle morphologies in Fig. 1 H, Fig. 2 E, and Fig. 3 E, slides were assigned blinded codes before immunostaining, imaged and analyzed, and then decoded after analysis. The LSM 880 microscope is housed in the Cell Sciences Imaging Facility located in and supported by the Beckman Center for Molecular and Genetic Medicine.

### Embryonic viability assays

Except where noted, embryonic viability, X-chromosome missegregation, and brood sizes were measured at 20°C for 12 individual worms of each genotype. Individually late L4 hermaphrodites were singled onto seeded NGM plates containing a small bacterial lawn. Individual worms were transferred daily (2×) to a fresh plate. Plates were scored 24 h after removal of the parent for the number of eggs and hatched larva; plates were scored again 3 days later to assess numbers of adult hermaphrodites and males.

### CRISPR generation of *tbb-2(E439K)* and *mei-2(A237T)* alleles

CRISPR editing was performed as described (Paix et al., 2015). sgRNA and PAM sites for all targets were selected using the CRISPOR website. A list of crRNAs and primers used are provided in Table S4. An injection mixture of target gene crRNA and repair oligo, co-CRISPR marker *dpy-10* repair oligo (Arribere et al., 2014, IDT), *dpy-10* crRNA (5′-GCT​ACC​ATA​GGC​ACC​ACG​AG-3′, IDT), trRNA (IDT), and Cas9-NLS nucleases (IDT) was injected into young adults of the appropriate genotypes. Appropriate edits were identified by PCR screening and confirmed by Sanger sequencing.

### Live *in utero* imaging

L4 larvae were incubated at 20°C overnight on seeded NGM plates, and 15–20 young adult hermaphrodites were picked into 1.5 μl of a worm soaking solution (20 mM Serotonin [H7752-1G; Sigma-Aldrich], 83 mM tetramisole hydrochloride [Levamisole, L9756; Sigma-Aldrich] and 10% tricaine [prepared in water and kept on ice]) on the bottom of #1.5 Ibidi 35 mm dish (50305805; Thermo Fisher Scientific). 0.75 μl of 0.1 μm polystyrene microspheres (08691; Polysciences) was added to the mixture and worms were left to immobilize for 10 min. A thin agarose pad (5% agarose in water and soaked in 40 μl of soaking solution briefly after preparation) was gently dried with a Kimwipe and then inverted and laid on top of the worms using tweezers to create a dish-worm-pad sandwich. A wet Kimwipe was rolled around the bottom of the Ibidi dish and the lid placed on top to create a humid chamber. The dish was placed on the top of an inverted spinning disk microscope and imaged from below. Meiotic oocytes before onset of ovulation were identified by bright field microscopy before initiating time-lapse fluorescence imaging.

Fluorescence imaging was performed at 20°C on a Nikon Ti-E Inverted spinning disk confocal microscope (Nikon Instruments) using a 60× PLAN APO oil objective (NA = 1.4) controlled using NIS Elements software (Nikon), RRID:SCR_014329. Movies of oocytes/embryos expressing GFP::TBA-2 and mCherry::H2B were captured using an Andor DU-897 X-6910 camera controlled using NIS Elements software (Nikon), RRID:SCR_014329. Z-series time-lapse images (Z-stack containing the entire oocyte, 2-μm step size; Laband et al., 2018) were collected at 12-s intervals. The 488 and 561 nm channels were imaged simultaneously at 5% and 10% power, respectively, at 250-ms exposure time. Focus was adjusted manually during time-lapse imaging.

### Analysis of meiosis I timing

To assess progression through meiosis I, we designated the appearance of microtubules around chromosomes following nuclear envelope breakdown to mark onset of meiotic spindle assembly (t = 0) and collected z-stacks every 12 s. Cell cycle stages and landmark events (illustrated in Fig. 7 A) were defined as previously described by pioneering live-imaging studies of *C. elegans* oocyte meiosis (Chuang et al., 2020). Metaphase is when chromosomes are fully congressed on bipolar spindles; anaphase A onset is when the spindle has shortened to its shortest length and bivalents have separated into homologs and begun moving toward opposite poles; anaphase B onset is when the chromosome masses have reached the outside of the spindle microtubules and the spindle starts to elongate; maximum extent of chromosome separation is when the spindle has reached maximum length and chromosome masses are separated by maximum distance, before actinomyosin ring contraction causes the spindle to bend away from the cortex; end of meiosis I is defined by polar body extrusion.

Chromosome separation over time and anaphase B chromosome separation velocity were analyzed as previously described (Li et al., 2023). For each spindle, distances between separating homologs (anaphase I) were measured in 3D in ImageJ, RRID:SCR_003070, by drawing a line connecting the centers of both chromosome masses and measuring the length of the line in μm; in cases where the spindle became bent and GFP::TBA-2-labelled spindle microtubules were clearly visible, a segmented line was drawn along the bent spindle to connect the two chromosome masses. Measurements were made every 24 s (2 frames) for a period of 200–250 s, until maximum extent of chromosome separation was achieved. We note that TBB-2-only spindles achieved maximum extent of chromosome separation in under 200 s. To represent anaphase chromosome separation over time, chromosome separation distances (every 24 s since anaphase A onset [t = 0]) for multiple spindles were averaged together to generate the cumulative line profiles shown in Fig. 7 E. Anaphase B chromosome separation velocities for each spindle (for the first 150 s in Fig. 7 F and for the entire anaphase B duration Fig. S5 D) were calculated as follows: the distance between chromosome masses at t 0 (anaphase B onset) was subtracted from distance between chromosome masses at the end point, and this value was divided by the elapsed time (in seconds) to obtain separation velocity in μm per second.

### GFP::TBA-2 and mCherry::H2B fixed fluorescence imaging

Worms were prepared, dissected and fixed as for immunofluorescence experiments. After 35 min in methanol, slides were washed twice in 1X PBST, mounted in mounting media (0.5% p-phenylenediamine, 20 mM Tris-Cl, pH 8.8, and 90% glycerol), and a coverslip (22 × 22 mm #1) was gently laid on top and then sealed with nail polish. Samples were imaged on a Zeiss LSM 880 Confocal microscope with a 63X oil (NA = 1.58) objective.

### MEI-1/2 signal distribution analysis

Analysis was performed in ImageJ, RRID:SCR_003070, using Sum projected images of spindles oriented roughly perpendicular to the Z axis. For each spindle, a “line” wide enough to capture the full width of the spindle was drawn along the long axis of the spindle, connecting the poles; pixel values at each position were extracted in ImageJ, RRID:SCR_003070, and recorded in Excel. Identical lines were drawn in an area of the cytoplasm adjacent to the spindle to extract background signal values, which were averaged to obtain mean background signal. For each position along the spindle, background-subtracted signal values were calculated, and signals were further internally normalized by dividing by the mean background-subtracted signal for the entire spindle. Finally, the normalized signal distribution for each spindle was then plotted against normalized spindle length (obtained by diving distances along the spindle by the maximum spindle length) in Excel to generate line profiles in Fig. 4 C and Fig. S3 C.

To compare distributions between genotypes, we generated an additional metric for each spindle, i.e., the average normalized signal for the central spindle (from 0.4 to 0.6 along the normalized spindle length).

### FRAP

FRAP experiments were performed on a Nikon Ti2 CREST spinning disk confocal microscope (Nikon Instruments) using a 60× PLAN APO oil objective (NA = 1.4) controlled using GATACA Systems ILAS Controller box. Worms were mounted with a set up similar to that used in live *in utero* imaging. Photobleaching was performed using a 473 nm laser at 5% laser power with 100-μs dwell time on an elliptical region of interest (ROI). Each frame was exposed for 300 ms with 1-s delay between acquisitions. Image analysis was performed using ImageJ, RRID:SCR_003070. Fluorescence signal was extracted every 3 s. If the spindle moved during movie acquisition, the ROI was manually moved to the approximate area of the bleached region. The raw fluorescence signal measurements were exported and analyzed in Excel. To track recovery, fluorescence signal measurements at each time point were subtracted from signal measurements at onset of bleaching (t = 0 s) and then normalized to the signal at the start of movie acquisition (t = −3 s). Recovery curves were generated in R studio.

### Statistical methods

Unless otherwise stated, two-tailed Mann–Whitney *P* values were calculated in GraphPad Prism 10, RRID:SCR_002798. Fisher’s exact tests were used to analyze contingency tables for categorical data using https://www.graphpad.com/quickcalcs/.

### Quantification of other spindle features

#### Spindle length

Analyzed in 3D using ImageJ, RRID:SCR_003070. For fixed immunofluorescence images in Fig. 5 B, the center of each pole was defined using the XYZ coordinates of the ASPM-1 signal, as it represents the point where the dense pole-associated microtubules terminate at the ends of the spindle. For the live images in Fig. 7 B and Fig. 8 A, the ends of spindles were estimated from GFP::TBA-2 fluorescence, with poles defined as the point where bright GFP::TBA-2 fluorescence terminates. Spindle lengths (in μm) were determined from the XYZ coordinates using the formula:

$$\sqrt{{\left(z2-z1\right)}^{2}+{\left(y2-y1\right)}^{2}+{\left(x2-x1\right)}^{2}}.$$

Quantification of spindle length in Fig. 5 B and Fig. 8 A were performed as blinded.

#### ASPM-1 and KLP-18 signal measurements

Image analysis was performed in ImageJ, RRID:SCR_003070, using sum-projected images of meiosis I spindles acquired identically at similar laser power. For each spindle, a sum projection of z-sections encompassing the full spindle was generated, an elliptical ROI was drawn around the pole in the channel of the sum projection, and the integrated density (mean pixel intensity x area) was recorded for the channel of interest. To calculate the background for each channel of interest, the same ROI was shifted to an area of the cytoplasm next to each pole and integrated density measurements were recorded. For each spindle, the background measurements were then subtracted from pole measurements, and the two background-subtracted poles were averaged together to obtain total signal. Each data point represents one spindle.

For KLP-18 normalized signal, the background-subtracted KLP-18 signal was divided by the background-subtracted tubulin signal to normalize for variability in staining.

#### Microtubule bundle length

Distances (in μm) from the base of microtubule bundle to mid-bivalent were measured in 3D using XYZ coordinates as for spindle length measurements. The base of the bundle is defined as a region of high microtubule density that colocalizes with high ASPM-1 signal in the area of the monopole; groups of microtubule bundles emanating from the base form lateral associations with a given bivalent. For each monopolar spindle, a line was drawn connecting the base to mid-bivalent for each bivalent, and all measurements were averaged together to obtain an average distance for a given spindle. For measurements of microtubule bundle lengths, slides were assigned blinded codes before immunostaining, imaged and analyzed, and then decoded after analysis.

#### Midspindle angle

Midspindle angles were measured using maximum intensity projections of spindles in which both poles were visible. The center of the spindle was identified as the area containing mCherry::H2B fluorescence corresponding to the congressed chromosomes. Midspindle angle was measured in ImageJ, RRID:SCR_003070, by drawing a line connecting the center of spindle to the center of each spindle pole. The resulting data were binned (5^°^ increments) and visualized in R studio.

### Online supplemental material

Fig. S1 shows that both TBB-1 and TBB-2 are readily incorporated into oocyte spindles throughout the meiotic cell cycle and that loss of TBB-1 exacerbates meiotic spindle assembly defects caused by *klp-18ts*. Fig. S2 shows that immunostaining experiments did not detect differences in KLP-18 localization between *TBB-1-only* and *TBB-2-only* oocyte spindles. Fig. S3 shows that β-tubulin isotype composition differentially affects association of katanin catalytic subunit MEI-1 with meiotic spindles. Fig. S4 shows that control, TBB-1-only, and TBB-2-only oocyte spindles exhibit similar extent and kinetics of recovery by FRAP analysis. Fig. S5 shows additional analysis of meiosis I timing in control, TBB-2-only, and TBB-1-only oocytes. Videos 1, 2, and 3 show time-lapse images of FRAP analysis in unarrested metaphase I oocyte spindles from control, TBB-2-only, and TBB-1-only worms. Videos 4, 5, and 6 show time-lapse images of oocyte meiosis I progression from live oocytes expressing GFP::TBA-2 to mark microtubules and mCherry::H2B to mark chromosomes. Table S1 shows quantification of progeny production, embryonic viability and male frequency for worms carrying *klp-18(or447ts)* and/or β-tubulin mutations. Table S2 shows quantification of progeny production, embryonic viability, and male frequency for worms carrying *klp-18(or447ts)*, *tbb-2(E439K)*, and/or β-tubulin substitution mutations. Table S3 shows quantification of progeny production, embryonic viability, and male frequency for worms carrying *klp-18(or447ts)*, *mei-2(A237T)*, and/or β-tubulin substitution mutations. Table S4 lists sgRNAs, repair templates, and genotyping primers for mutant alleles generated by CRISPR-Cas9 in this study.

## Supplementary Material

Table S1 shows quantification of progeny production, embryonic viability, and male frequency for worms carrying *klp-18(or447ts)* and/or β-tubulin mutations.

Table S2 shows quantification of progeny production, embryonic viability, and male frequency for worms carrying *klp-18(or447ts), tbb-2(E439K)*, and/or β-tubulin substitution mutations.

Table S3 shows quantification of progeny production, embryonic viability, and male frequency for worms carrying *klp-18(or447ts), mei-2(A237T)*, and/or β-tubulin substitution mutations.

Table S4 shows lists sgRNAs, repair templates, and genotyping primers for mutant alleles generated by CRISPR-Cas9 in this study.

## Data Availability

Materials used in this study are available upon reasonable request from A.M. Villeneuve. Images and spreadsheets have been deposited in BioStudies Database Accession number: S-BSST3183 (https://doi.org/10.6019/S-BSST3183).
