# Supplementary material for: Differential contributions of β-tubulin isotypes to acentrosomal oocyte meiosis in C. elegans
Source: J Cell Biol. 2026 Aug 10;225(10):e202511199. doi: 10.1083/jcb.202511199 (PMC13455592; doi:10.1083/jcb.202511199)
Supplement: Supplementary file 1 [file jcb_202511199_tables1.docx]

**Table S1 Experiment 1**. Progeny production by worms carrying *klp-18(or447ts)* and/or β-tubulin mutations.

| **Maternal genotype** | **% hatching (20°)**  **(no. of embryos)** | **% viability (20°)**  **(no. of adults)** | **% Males**  **(no. of adults)** | **Average no. of eggs laid (no. of worms)** |
| --- | --- | --- | --- | --- |
| *tbb-1(0)* | 98.5 (2526) | 96.7 (2440) | 0.1 (2440) | 253 (10) |
| *tbb-2(0)* | 60.0 (1908) | 52.1 (995) | 2.0 (995) | 127 (15) |
| *klp-18ts* | 53.4 (1959) | 45.0 (881) | 7.4 (881) | 163 (12) |
| *tbb-1(0); klp-18ts* | 2.1 (2091) | 0.5 (10) | 50 (10) | 209 (10) |
| *tbb-2(0); klp-18ts** | 26.0 (1672) | 13 (215) | 7.0 (215) | 139 (12) |
| *TBB-1-only; klp-18ts* | 60.3 (1997) | 51.3 (1025) | 5 (1025) | 166 (12) |
| *TBB-2-only; klp-18ts* | 3.2 (1766) | 1.2 (21) | 24 (21) | 147 (12) |

* The phenotype of *tbb-2(0); klp-18ts* double mutant represents the combined effects of two deleterious mutations that individually impair viability (rather than synthetic lethality as for *tbb-1(0); klp-18ts*); further, the modest excess lethality arises during larval development, indicating that it is not caused by meiotic defects and thus is outside the scope of the present study.

**Table S1 Experiment 2**. Progeny production by worms carrying *klp-18(or447ts)* and/or β-tubulin substitution mutations.

| **Maternal genotype** | **% hatching (20°)**  **(no. of embryos)** | **% viability (20°)**  **(no. of adults)** | **% Males**  **(no. of adults)** | **Average no. of eggs laid (no. of worms)** |
| --- | --- | --- | --- | --- |
| *TBB-1-only* | 98.4 (2152) | 98.3 (2116) | 0.1 (2116) | 307 (7) |
| *TBB-2-only* | 98.2 (2143) | 98.0 (2101) | 0.1 (2101) | 306 (7) |
| *klp-18ts* | 75.3 (821) | 68.6 (563) | 2.8 (563) | 137 (6) |
| *TBB-1-only; klp-18ts* | 44.5 (1199) | 42.0 (504) | 6.9 (504) | 150 (8) |
| *TBB-2-only; klp-18ts* | 5.3 (1343) | 1.9 (26) | 30.8 (26) | 168 (8) |
