# Supplementary material for: Differential contributions of β-tubulin isotypes to acentrosomal oocyte meiosis in C. elegans
Source: J Cell Biol. 2026 Aug 10;225(10):e202511199. doi: 10.1083/jcb.202511199 (PMC13455592; doi:10.1083/jcb.202511199)
Supplement: Supplementary file 2 [file jcb_202511199_tables2.docx]

**Table S2 Experiment 1**. *tbb-2(E439K)* suppresses meiotic defects of *TBB-2-only; klp-18(or447ts)*

| **Maternal genotype** | **% hatching**  **(no. of embryos)** | | **% viability**  **(no. of adults)** | | **% Males**  **(no. of adults)** | | **Average no. of eggs laid (no. of worms)** | |
| --- | --- | --- | --- | --- | --- | --- | --- | --- |
|  | **20°** | **25°** | **20°** | **25°** | **20°** | **25°** | **20°** | **25°** |
| *klp-18ts* | 75.6 (1744) | 0.8  (360) | 73.3  (1279) | 0.3  (1) | 2.3 (1279) | 100  (1) | 159 (11) | 60 (6) |
| *TBB-2-only; klp-18ts* | 3.6  (2341) | 0.9  (774) | 1.8  (42) | 0.1  (1) | 21  (42) | 0  (1) | 195 (12) | 129 (6) |
| *tbb-2(E439K), TBB-2-only; klp-18ts* | 82.7  (2001) | 11.9  (494) | 78.2  (1564) | 9  (44) | 1.4  (1564) | 2.3  (44) | 167  (12) | 82  (6) |

**Table S2 Experiment 2**. *tbb-2(E439K)* suppresses meiotic defects of *TBB-2-only; klp-18(or447ts).* Progeny production by worms carrying *klp-18(or447ts), tbb-2(E439K)* and/or β-tubulin substitution mutations

| **Maternal genotype** | **% hatching (20°)**  **(no. of embryos)** | **% viability (20°)**  **(no. of adults)** | **% Males**  **(no. of adults)** | **Average no. of eggs laid (no. of worms)** |
| --- | --- | --- | --- | --- |
| *TBB-2-only* | 98.4 (1860) | 98.4 (1830) | 0.1 (1830) | 266 (6) |
| *tbb-2(E439K)* | 98.9 (1446) | 98.9 (1430) | 0.0 (1430) | 206 (7) |
| *klp-18ts* | 87.3 (895) | 84.5 (756) | 2.0 (756) | 128 (7) |
| *TBB-2-only; klp-18ts* | 6.6 (1024) | 1.8 (18) | 5.6 (18) | 146 (7) |
| *tbb-2(E439K), TBB-2-only; klp-18ts* | 81. 6 (778) | 77.2 (601) | 1.5 (601) | 156 (5) |
