# Supplementary material for: Differential contributions of β-tubulin isotypes to acentrosomal oocyte meiosis in C. elegans
Source: J Cell Biol. 2026 Aug 10;225(10):e202511199. doi: 10.1083/jcb.202511199 (PMC13455592; doi:10.1083/jcb.202511199)
Supplement: Supplementary file 3 [file jcb_202511199_tables3.docx]

**Table S3 Experiment 1**. Suppression of meiotic defects of *TBB-2-only; klp-18(or447ts)* by *mei-2(A237T)* mutation causing reduced katanin activity.

| **Maternal genotype** | **% hatching (20°)**  **(no. of embryos)** | **% viability (20°)**  **(no. of adults)** | **% Males**  **(no. of adults)** | **Average no. of eggs laid (no. of worms)** |
| --- | --- | --- | --- | --- |
| *mei-2(A237T)* | 99.1 (3443) | 99.1 (3413) | 0.0 (3413) | 287 (12) |
| *klp-18ts* | 82.6 (1965) | 78.2 (1537) | 2.3 (1537) | 164 (12) |
| *TBB-2-only; klp-18ts* | 4.0 (2190) | 1 (21) | 19 (21) | 183 (12) |
| *mei-2(A237T); TBB-2-only; klp-18ts* | 74.8 (2304) | 66.1 (1523) | 0.7 (1523) | 192 (12) |

**Table S3 Experiment 2**. Suppression of meiotic defects of *TBB-2-only; klp-18(or447ts)* by *mei-2(A237T)* mutation. Progeny production by worms carrying *klp-18(or447ts), mei-2(A237T)* and/or β-tubulin substitution mutations

| **Maternal genotype** | **% hatching (20°)**  **(no. of embryos)** | **% viability (20°)**  **(no. of adults)** | **% Males**  **(no. of adults)** | **Average no. of eggs laid (no. of worms)** |
| --- | --- | --- | --- | --- |
| *mei-2(A237T)* | 99.8 (1683) | 99.8 (1679) | 0.1 (1679) | 281 (6) |
| *klp-18ts* | 77.0 (688) | 76.5 (526) | 3.2 (526) | 115 (6) |
| *mei-2(A237T); klp-18ts* | 59.6 (747) | 58.8 (439) | 1.6 (439) | 107 (6) |
| *TBB-2-only; klp-18ts* | 3.8 (814) | 2.1 (17) | 35.3 (17) | 136 (6) |
| *mei-2(A237T); TBB-2-only; klp-18ts* | 52.1 (716) | 49.2 (352) | 1.7 (352) | 119 (6) |
