# Supplementary material for: Differential contributions of β-tubulin isotypes to acentrosomal oocyte meiosis in C. elegans
Source: J Cell Biol. 2026 Aug 10;225(10):e202511199. doi: 10.1083/jcb.202511199 (PMC13455592; doi:10.1083/jcb.202511199)
Supplement: Supplementary file 4 [file jcb_202511199_tables4.docx]

**Table S4:** Reagents for CRISPR-Cas9 editing

| Allele | Target gene | Sequence description | Sequence |
| --- | --- | --- | --- |
| *me196 (E439K)* | *tbb-2* | sgRNA | actgccgaagacgacgtcga |
|  |  | Repair template | CAACAATACCAAGAAGCAACTGCCGAAGACGACGTCGACGGATACGCTAAGGGAGAAGCTGGAGAGACTTACGAATCGGAGCAA |
|  |  | Forward primer | CCTTCGTTGGAAACTCGACC |
|  |  | Reverse primer | TCAAGCGTATCTCAGAGCAG |
|  |  |  |  |
| *me198 (A237T)* | *mei-2* | sgRNA | tcggcgaattggagtcgatg |
|  |  | Repair template | GATTTGCATCCACCAAAACTCGGCGAATTGGAGTCGACGTTGTTGCTGAAGAGAGAGCTGCTAAAACAACAGAGTGCATTCACAATTTTCGCAAAATAGTT |
|  |  | Forward primer | CTTGGTGTCTCAACTCGTGC |
|  |  | Reverse primer | AATCAGTTCCTCCACGAGG |
